# GEODE: an *in silico* tool that translates *in vitro* to *in vivo* predictions of tuberculosis antibiotic combination efficacy

**DOI:** 10.3389/fphar.2025.1639673

**Published:** 2025-10-17

**Authors:** Maral Budak, Mariana Pereira Moraes, Talia Greenstein, Pauline Maiello, H. Jacob Borish, Harris B. Chishti, Kara Kracinovsky, Mark Rodgers, Jaime Tomko, Philana Ling Lin, JoAnne L. Flynn, Bree B. Aldridge, Denise Kirschner

**Affiliations:** ^1^ Department of Microbiology and Immunology, University of Michigan Medical School, Ann Arbor, MI, United States; ^2^ Department of Molecular Biology and Microbiology, Tufts University School of Medicine, Boston, MA, United States; ^3^ Graduate School of Biomedical Sciences, Tufts University School of Medicine, Boston, MA, United States; ^4^ Department of Microbiology and Molecular Genetics, University of Pittsburgh, Pittsburgh, PA, United States; ^5^ Department of Pediatrics, Children’s Hospital of the University of Pittsburgh of UPMC, Pittsburgh, PA, United States; ^6^ Department of Biomedical Engineering, Tufts University School of Engineering, Medford, MA, United States

**Keywords:** PK/PD, drug rankings, DiaMOND, checkerboard, GranSim

## Abstract

**Introduction:**

Tuberculosis (TB) remains the primary cause of death due to infectious disease in the world. TB, while treatable, requires an extended course of multiple antibiotics, taking 6–9 months, and many antibiotic regimens have deleterious side effects. Treatment is complicated by co-infection, emerging drug resistance, and compliance issues; accordingly, the identification of new and optimal regimens has been a recent focus. Rodent models of TB (e.g., mouse, rabbit) do not mimic some severe pathologies well, while nonhuman primate models are costly. Several computational and *in vitro* tools have been developed to explore drug regimen design and efficacy for TB, each providing insight into human disease dynamics.

**Methods:**

Here we briefly review existing tools and introduce a novel, integrated approach combining *in vitro* predictions of drug pharmacokinetics, pharmacodynamics and drug-drug interactions with a granuloma-scale computational model (*GranSim*). Our method captures *in vivo* dynamics to test how well systematic *in vitro* data predict granuloma-scale outcomes such as CFU burden and sterilization time. To evaluate *in vitro* measurements under various growth conditions and to compare to clinical and experimental datasets, we simulated five well-known regimens in our pipeline: HRZM, BPaMZ, RMZE, BPaL and HRZE.

**Results:**

We find that *in vitro* measurements of antibiotic regimen pharmacodynamics under specific growth conditions can be used to simulate virtual granulomas consistent with low-burden human and primate granulomas.

**Discussion:**

This work provides a novel tool that can be used to quickly and efficiently evaluate drug regimens for TB.

## Introduction

Pulmonary tuberculosis (TB) is caused by the inhalation of *Mycobacterium tuberculosis* (Mtb) leading to infection within lungs. This leads to formation of granulomas, hallmark lesions composed of immune cells, Mtb and necrotic tissue (referred to as caseum due to its cheese-like appearance). Granulomas are heterogeneous within a given host, giving rise to diverse microenvironments for Mtb (Lenaerts et al., 2015). To survive within these environments, Mtb adapts to these conditions by adjusting metabolic activities, decreasing replication rates or increasing lipid production, etc. (Meena and Rajni, 2010; Gengenbacher and Kaufmann, 2012). Such adaptations lead to various Mtb phenotypes even within a single granuloma (Lenaerts et al., 2015). Unfortunately, Mtb can become more tolerant to anti-Mtb antibiotics due to non-adherence and microenvironment heterogeneity (Lenaerts et al., 2015; Dartois and Rubin, 2022; Sarathy et al., 2018; Sarathy and Dartois, 2020).

Treatment remains challenging despite effective drugs due to poor access to consistent medical treatment, side effects of the drugs and other obstacles to treatment; hence TB remains one of the deadliest infectious diseases in the world with 1.25 million deaths per year (World Health Organization, 2024). This challenge is also related to the complexities of granulomas (Sarathy et al., 2018; Sarathy and Dartois, 2020) and varied metabolic phenotypes of Mtb (Dhar et al., 2016). Granulomas provides a spatially heterogeneous physiological barrier that prevents uniform distributions of administered compounds (Sarathy et al., 2016; Pienaar et al., 2015a; Pienaar et al., 2017). Moreover, the pharmacokinetic (PK) variability within populations leads to different levels of antibiotic exposure within granulomas (Cicchese et al., 2020a). As Mtb have evolved various phenotypes to survive within granulomas, treatment necessitates compounds with diverse pharmacodynamic (PD) mechanisms of action (Kerantzas and Jacobs, 2017). Moreover, extremely slow-growing bacteria like Mtb cannot easily be detected and killed by antibiotics, and Mtb can acquire antibiotic resistance over the course of treatment (Johnson et al., 2006). Combination therapy is required for treatment of Mtb infection to target various Mtb phenotypes, facilitate penetration of antibiotics within all types of granuloma lesions and prevent acquisition of antibiotic resistance (Kerantzas and Jacobs, 2017).

The current standard TB treatment regimen consists of four antibiotics (isoniazid, rifampicin, pyrazinamide, and ethambutol; HRZE) over 6–9 months (Nahid et al., 2016). This long treatment time with multiple antibiotics that have many side effects leads to adherence issues, which can lead to unfavorable outcomes (Tola et al., 2019). Shortening treatment times and developing more patient-friendly and potent combination regimens are urgently needed. To this end, recent research efforts have focused on discovering novel regimens for TB treatment to replace HRZE as the standard regimen. Animal models and clinical trials are informative and useful to assess drug efficacy. However, they are costly and time-consuming, hence they are not feasible to efficiently test multiple, different combination regimens. Moreover, 90% of clinical trials fail (Sun et al., 2022), emphasizing the need for initial screening methods to inform clinical trials for faster, more efficient, and cost-effective drug development efforts.

Typically, mouse models and *in vitro* studies have been used to identify drug doses and regimens (De Groote et al., 2011; Cokol et al., 2017). However, mathematical and computational modeling is another tool that has had success predicting drug regimen design that offsets several of the challenges that face *in vivo* or *in vitro* studies (Pienaar et al., 2015a; Cicchese et al., 2017; Budak et al., 2023). We seek to leverage *in vitro, in vivo,* and *in silico* models into a pipeline that integrates these different tools for better, faster, and more robust efficacy predictions of new and optimal regimen designs prior to clinical trial.

### Current drug screening approaches

#### *In vivo* models

There are several animal models for TB that have been used for TB drug discovery and development efforts, such as mouse, rabbit and nonhuman primates (NHPs) (Zhan et al., 2017). Mouse models are advantageous and more manageable due to their low cost and low maintenance (Zhan et al., 2017; Shi et al., 2011). However, their immune response to Mtb differs from that of humans in several ways, notably that standard mouse models do not produce well-formed granulomas and if they do, lesions are non-necrotic. This is unlike the spectrum of heterogeneous, often necrotic granulomas observed in humans (Zhan et al., 2017; Shi et al., 2011). TB disease in rabbits resembles human TB as they develop lung necrotic granulomas (Gupta and Katoch, 2005). However, assessing their immunological response is challenging due to the lack of relevant immunological reagents (Gupta and Katoch, 2005). NHP models have similar immunological responses as compared to humans upon Mtb infection and develop a full spectrum of granuloma types, including necrotic granulomas like humans (Scanga and Flynn, 2014). This makes them one of the most suitable models for TB research. However, NHPs require extensive veterinary and facility costs and produce results slowly, thus limiting their feasibility (Scanga and Flynn, 2014).

#### *In vitro* models

There are a variety of *in vitro* assays available to assess drug efficacies in Mtb; these may use solid or liquid medium and assess growth inhibition or bactericidal activities (Sarathy et al., 2018; Cokol et al., 2017; Larkins-Ford et al., 2021). Hollow fiber system models mimic the *in vivo* PK profiles of anti-TB drugs, which ensures that Mtb is exposed to antibiotic concentrations similar to those *in vivo* and allows for PD predictions that are representative of *in vivo* conditions (Gumbo et al., 2004; Gumbo et al., 2007a; Gumbo et al., 2007b). Still, these approaches lack host immunological responses that significantly alter treatment responses.

#### *In silico* models

Researchers have used mathematical, statistical and computational modeling to accelerate drug discovery efforts in a cost-efficient way. Empirical approaches use data-driven models to assess how certain outcomes are affected by predictive variables within a set of experiments and to predict outcomes of circumstances that have not been experimented before. Examples for these types of studies include meta-analyses (Berg et al., 2022; Bonnett et al., 2017), linear and nonlinear mixed effects modeling studies (Berg et al., 2022; Ernest et al., 2023; Ngo et al., 2024) or machine learning approaches (Clemens et al., 2019; Strydom et al., 2025). These studies use datasets from clinical trials or *in vivo* studies to predict better treatment regimens for TB. This approach requires simpler models; however, these models lack mechanistic reasoning behind observations and cannot reliably extrapolate beyond the realm of the data that was used to calibrate them.

Mechanistic models, on the other hand, capture complex mechanisms and dynamics of biological processes to make realistic predictions of interventions or predict alternative approaches to improve intervention outcomes (Chakraborty, 2017). Also, to include effects of the immune response on drug treatment outcomes, the use of models that capture host-responses can be more predictive of *in vivo* dynamics.

Over the past few decades, quantitative systems pharmacology has become a burgeoning field that has contributed to a number of drug studies for diseases such as various types of cancers (Wang et al., 2021; Nikfar et al., 2023), cardiovascular diseases (Cheng et al., 2022; Fang et al., 2019), infectious diseases (Liu et al., 2018; Rao et al., 2023) etc. Mechanistic models have been used for over 2 decades to study TB (Kirschner et al., 2017; Nanda et al., 2023). To efficiently explore complexities of Mtb-host interactions specifically at the granuloma scale, we previously developed a computational pipeline that mechanistically simulates granuloma treatment with various combinations of antibiotics mechanistically (Pienaar et al., 2015a; Pienaar et al., 2017; Budak et al., 2023; Pienaar et al., 2015b; Budak et al., 2024). This pipeline utilizes *GranSim*, a multi-scale mechanistic, hybrid model that combines agent-based modeling with discretized both partial and ordinary differential equations (ODEs), that simulates host-immune responses after Mtb infection within lungs, to generate a wide variety of granuloma types (Segovia-Juarez et al., 2004; Ray et al., 2009). We incorporated PK/PD dynamics into *GranSim* to study the spatial and temporal dynamics of antibiotics within granulomas (Pienaar et al., 2015a). *GranSim* allows us the ability to simulate thousands of treatment regimens on the same large library of virtual granulomas, validate our results against datasets from clinical trials and animal studies, and predict which mechanisms drive granuloma outcomes or responses to treatment (Budak et al., 2023; Budak et al., 2024).

#### Drug-drug interaction studies

Given that TB treatment requires combination therapy, drug-drug interactions may significantly affect the efficacy of multi-drug regimens, as some combinations are synergistic while others are antagonistic (Cokol et al., 2017). Checkerboard assays are widely used to evaluate drug–drug interactions by systematically measuring drug-dose combinations (Michaud, 1996). A pairwise checkerboard assay is typically performed in a microtiter plate configured as a two-dimensional matrix, where one drug is serially diluted across the rows (x-axis) and the second drug is diluted down the columns (y-axis). Drug interactions are often visualized using isobologram plots connecting concentration pairs that produce equivalent levels of biological response, such as 50% growth inhibition. The shape of these contours provides qualitative insight into the nature of the interaction: linear contours typically indicate additivity, whereas concave and convex contours suggest synergy and antagonism, respectively. Quantitative interaction assessment is commonly performed using the fractional inhibitory concentration index (FICI), which compares the potency of each drug in combination to its potency when used alone. To assess drug interactions across a wide range of potential TB treatment combinations, checkerboard assays are often impractical due to their high demand for both experimental resources and manual effort. As a result, alternative methods have been developed to evaluate drug interactions more efficiently and at a greater scale. Computational approaches, such as INDIGO (INferring Drug Interactions using chemo-Genomics and Orthology) (Chandrasekaran et al., 2016; Ma et al., 2019), leverage chemogenomic data to predict fractional inhibitory concentrations (FICs) across large datasets. DiaMOND (Diagonal Measurement of N-way Drug Interactions) is an experimental and analytical approach that streamlines the checkerboard assay by focusing on the most informative portions of the dose-response surface. Rather than measuring every possible drug combination in a full checkerboard, DiaMOND uses single-drug dose responses along the axes and combination dose responses along the diagonal. This geometric framework allows for accurate estimation of interaction effects while significantly reducing experimental workload (Cokol et al., 2017; Larkins-Ford et al., 2021).

Although these *in silico* and *in vitro* studies can assess drug PK/PD and drug-drug interactions, respectively, they individually lack the ability to capture the role of host immunity and heterogeneous Mtb phenotypes present *in vivo*, both of which likely impact the course of treatment.

### GEODE: integration of tools to predict drug efficacy

**FIGURE 1:**
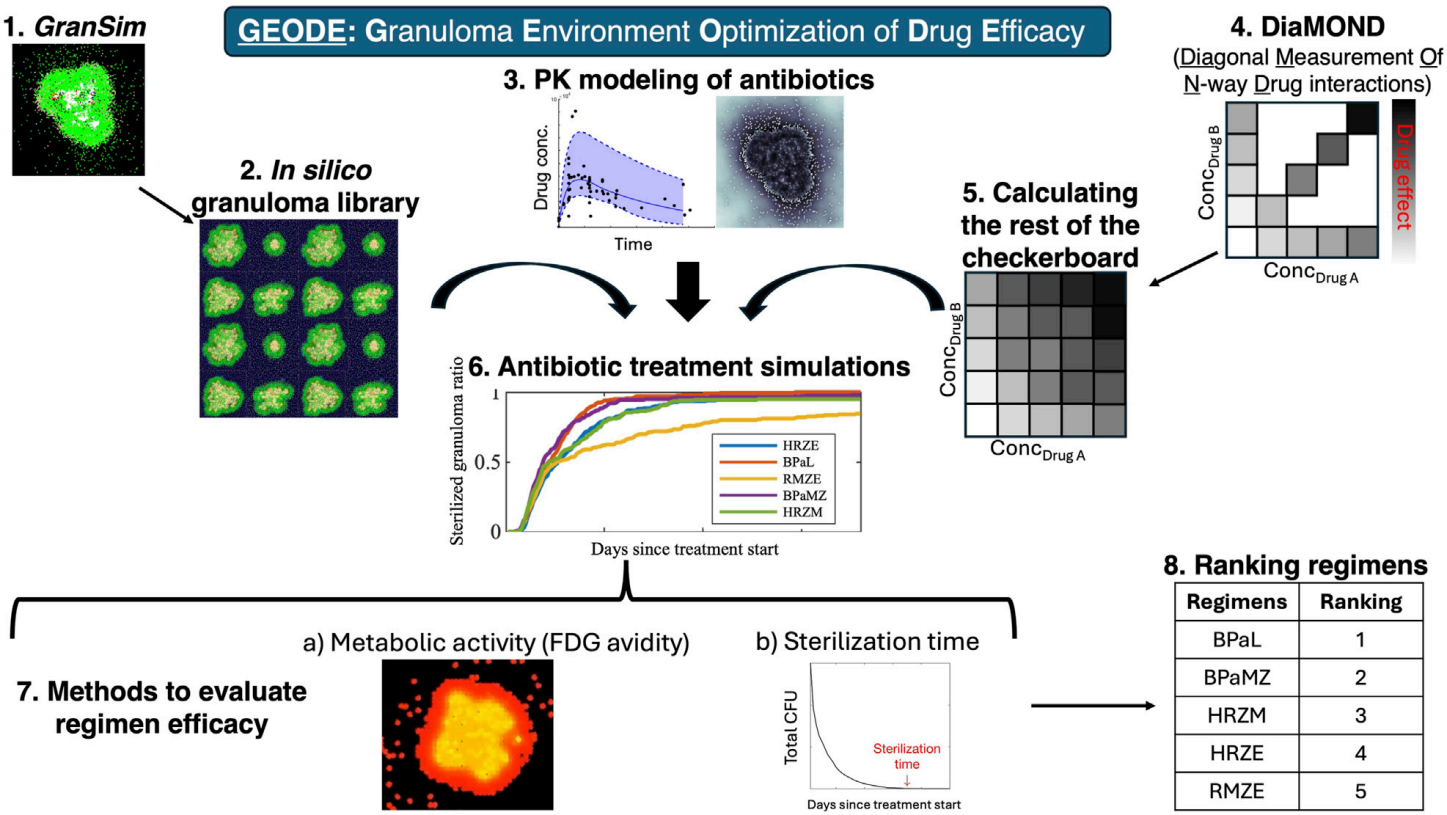
GEODE (Granuloma Environment Optimization of Drug Efficacy) pipeline. GEODE starts with (1) *GranSim*, our computational model of lung granulomas during pulmonary TB, which we use to create (2) an *in silico* granuloma library and incorporate (3) a PK model of antibiotics. Meanwhile, we perform (4) *In vitro* experiments, where we evaluate efficacies of single drugs and equipotent combinations against Mtb in various stress conditions, i.e., we generate the x- and y-axis and the diagonal of a standard checkerboard assay. We developed a methodology to (5) calculate the rest of the checkerboard, which we (6) included in *GranSim*. We then (7) performed antibiotic treatment simulations using our *in silico* granuloma library, PK model, and *in vitro* (DiaMOND-based) checkerboards. We (8) evaluated regimen efficacies by **(a)** measuring metabolic activity (FDG avidity) of granulomas before and after the treatment and **(b)** sterilization time of granulomas, i.e., time needed to kill all bacteria within granulomas. We (9) ranked regimens based on their sterilization times.

To predict regimen efficacies for TB treatment and inform clinical trials, we consider the following complexities occurring during Mtb infection: spatial heterogeneities, Mtb phenotypic tolerance of drugs due to phenotype, drug-drug interactions, host-pathogen interactions, the host immune responses, and the effect of bacterial burden per granuloma (whereby density of bacteria affects PD, also known as inoculum effect in *in vitro* studies). In previous studies, we used our *GranSim*-PKPD model and tracked drug action using bactericidal assays performed in nutrient-rich conditions, macrophage assays or caseum mimics from various studies, together with INDIGO predictions for drug-drug interactions (Pienaar et al., 2015a; Pienaar et al., 2017; Budak et al., 2023; Pienaar et al., 2015b; Budak et al., 2024; Cicchese et al., 2021). Here, we improve our model of drug action by incorporating DiaMOND measurements performed under different *in vitro* growth conditions that model stressors that Mtb encounter in distinct granuloma microenvironments. These conditions have been shown to be predictive of *in vivo* and clinical outcomes, thereby improving our efficacy predictions (Larkins-Ford et al., 2021; Larkins-Ford et al., 2022). Further, we provide a methodology to calculate bactericidal activity, completely filling in the checkerboards, building on the “diagonal” of DiaMOND checkerboards, and single-drug responses. This methodology enables us to elucidate the picture of multi-scale drug-drug interactions and to assess optimal dosages to find combination regimens with the highest overall bactericidal activity, which, practically, can provide promising, mechanistically founded predictions to investigate in future studies. We name our new method GEODE: **G**ranuloma **E**nvironment **O**ptimization of **D**rug **E**fficacy (Figure 1).

## Results

In this study, our goal is to improve our ability to predict successful regimens for clinical trial evaluation by combining different drug regimen screening approaches. Specifically, we calibrate GEODE with *in vitro* measurements to explore optimal dosage of the commonly-prescribed front-line and last-resort anti-Mtb drugs Bedaquiline (BDQ), Ethambutol (EMB), Isoniazid (INH), Linezolid (LZD), Moxifloxacin (MXF), Pretomanid (PTM), Pyrazinamide (PZA), and Rifampicin (RIF) (Larkins-Ford et al., 2021; Chahine et al., 2014; Bloch et al., 1954; Paton et al., 2023; Steele and Des Prez, 1988; Doster et al., 1973; Gillespie, 2016; Gils et al., 2022; Cox and Ford, 2012). Using our *in silico* model of granuloma formation and treatment, *GranSim*, we evaluate PK and PD drug-drug interactions of these antibiotics against Mtb grown in different *in vitro* stress conditions. Specifically, we (1) capture the effect of bacterial burden per granuloma (i.e., inoculum effect in *in vitro* studies) within *GranSim*, (2) map *in vitro* growth conditions to PD of drugs within *GranSim* to allow for nuanced simulation of bactericidal activity based on the metabolic state of each Mtb within virtual granulomas. To validate our approach, we then (3) virtually reproduce previously established efficacies and rankings of multiple regimens studied in (i) NHP studies and (ii) clinical trials by simulating granulomas under analogous conditions.

### Capturing the effect of bacterial burden within GranSim

**FIGURE 2:**
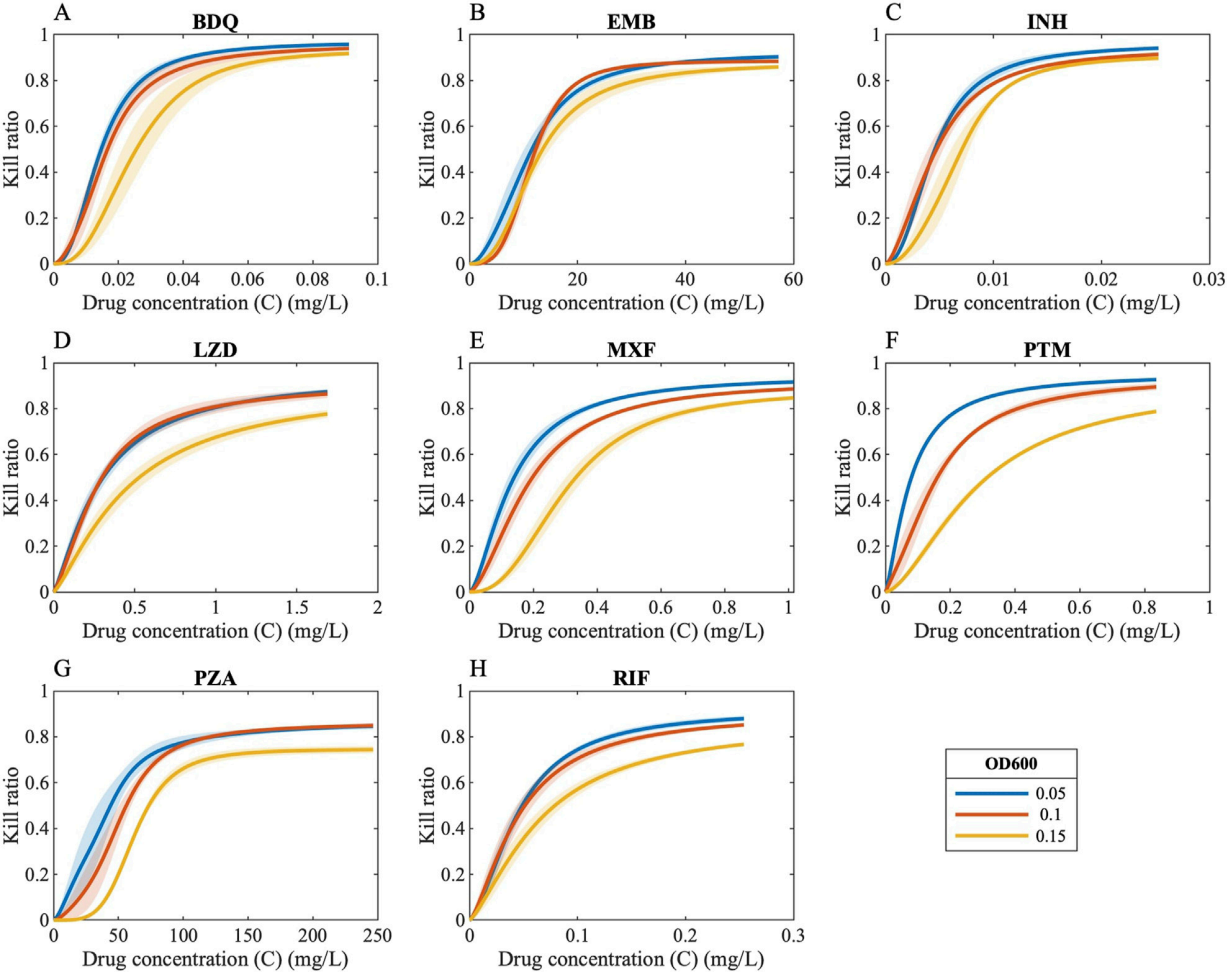
Drug concentrations versus kill ratios. Ratio of killed bacteria in the form of Hill curves as a function of drug concentration when antibiotics are administered to bacteria with varying bacterial loads [OD 600 values of 0.05 (blue curves), 0.1 (orange curves) and 0.15 (yellow curves)] [**(A)** BDQ: Bedaquiline, **(B)** EMB: Ethambutol, **(C)** INH: Isoniazid, **(D)** LZD: Linezolid, **(E)** MXF: Moxifloxacin, **(F)** PTM: Pretomanid, **(G)** PZA: Pyrazinamide, **(H)** RIF: Rifampicin].

Bacterial susceptibility to antibiotics can vary with bacterial burden, with higher bacterial burden potentially reducing drug efficacy (also called as “inoculum effect” in *in vitro* studies) (Brook, 1989), a phenomenon that has also been observed in *Mycobacterium tuberculosis* (Mtb) (Kirby and Dubos, 1947). To quantify and map the effect of Mtb burden, we conducted *in vitro* experiments to evaluate the bactericidal activity of antibiotics under a range of starting Mtb density. From these experiments, we generated pharmacodynamic (PD) data, modeled as Hill curves, which describe the drug-induced bacterial kill (measured as kill ratio in the GranSim framework; see Methods) as a function of antibiotic concentration for each bacterial load and drug tested (Figure 2).

**FIGURE 3:**
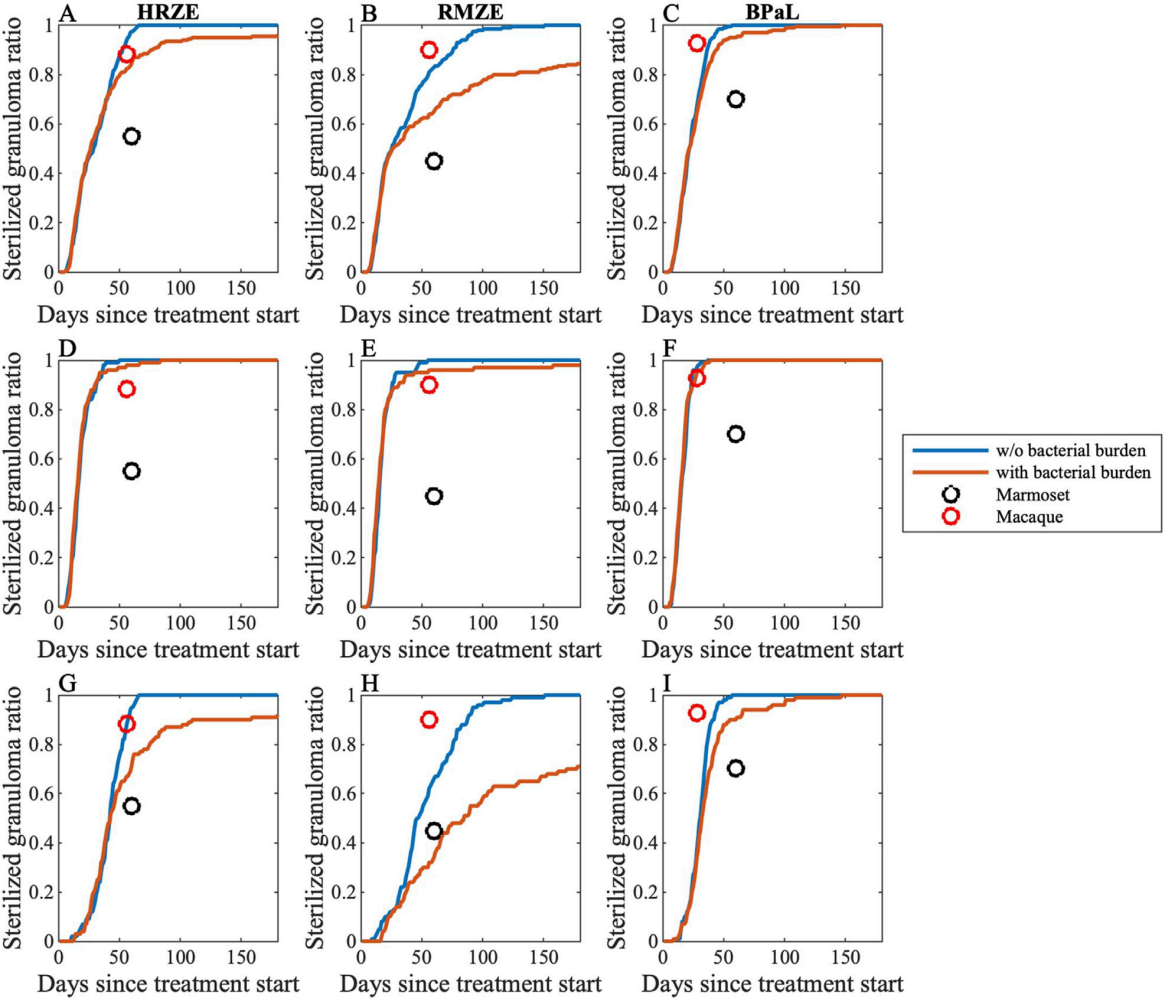
Sterilization curves of three different multi-drug regimens comparing with and without simulating effects of bacterial burden (see Equations 25–30). We compare simulations to two different NHP granuloma datasets. Sterilization curves of **(A,D,G)** HRZE, **(B,E,H)** RMZE and **(C,F,I)** BPaL considering **(A–C)** all granulomas, **(D–F)** low-CFU granulomas and **(G–I)** high-CFU granulomas. Each panel shows sterilization curves that represent the percentage of sterile virtual granulomas across time both with (red) and without (blue) simulating effects of bacterial burden. Black and red markers represent sterilized granuloma ratios from marmosets (Budak et al., 2024) and macaques (Budak et al., 2023; Michael CT et al., 2025), respectively.

We then translated this information into a pharmacokinetically relevant drug effect metric (E_PK_), which was fitted to exponential decay curves, capturing how E_PK_ declines with increasing bacterial load (see Methods). As anticipated, incorporating an effect of varying levels of bacterial burden into the model resulted in a slower predicted rate of bacterial sterilization (Figure 3).

To validate our model capturing bacterial burden effect, we compare simulated sterilization curves to granuloma-scale data from marmosets (Budak et al., 2024) and macaques (Budak et al., 2023; Michael CT et al., 2025). We calculate *sterilized granuloma ratios*, the portion of a set of granulomas that sterilized, for both marmoset and macaque datasets, pooling granulomas from different animals and assuming a limit of detection as five colony forming units (CFU) per granuloma and, for some analyses, grouping granulomas by their pre-treatment CFU burden. We have used between 5 and 10 CFU as a threshold for our recent published works on granuloma modeling at all scales. This range is based on the calculation from NHP granulomas that are plated. This accounts for the amount of media that the granuloma is homogenized in. The model is flexible and the results are consistent within this range of CFU as guided by the NHP data (Lin et al., 2016). Additionally, consider that CFU is calculated as CFU/mL = (Number of Colonies Counted × Dilution Factor)/Volume Plated (in mL). So in this case, if we find 1 colony on the neat (most concentrated) plate: CFU/mL = (1 colony*1)/.2 mL = 5 CFU/mL => 5 CFU/granuloma in the ideal case. So both experimentally and theoretically five is a solid choice.

As marmosets tend to have progressive TB disease and also have many granulomas with uncontrolled CFU growth (Via et al., 2013) (see Figure 10B for GranSim vs marmoset CFU counts), we see that marmoset sterilized granuloma ratios best-match those from only high-CFU virtual granulomas (see Methods and Figure 10 for details of high- and low-CFU granulomas). We provide evidence that sterilized granuloma ratios from marmosets fit well to *GranSim* when we include representation of an effect of bacterial burden (Figures 3G–I). Further, sterilized granuloma ratios from macaques best match to low-CFU virtual granulomas, as most cynomolgus macaque granulomas tend to have controlled CFU growth (Maiello et al., 2018; Flynn et al., 2017). Here, we show that sterilized granuloma ratios from macaques agree with sterilization curves from low-CFU granulomas when we represent the effect of bacterial burden in our model (Figures 3D–F).

### Generating the entire checkerboard from DiaMOND data

**FIGURE 4:**
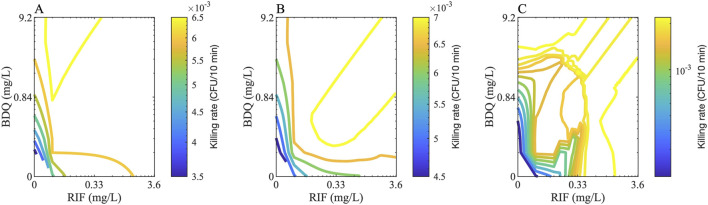
Checkerboard examples for two drugs. Example checkerboards for two drugs, Bedaquiline (BDQ) and rifampicin (RIF), that shows the rate of killing of **(A)** extracellular, **(B)** intracellular and **(C)** nonreplicating bacteria for various concentrations of both drugs. The color of the contours indicates the killing rate in one *GranSim* timestep (i.e., 10 min).

We developed a methodology to reconstruct the full checkerboard using the *in vitro* data measured via DiaMOND, enabling the estimation of bactericidal effects across all drug concentration combinations. This process involves three main steps: (1) converting growth-inhibition-based Hill parameters into bactericidal parameters; (2) deriving new Hill equation parameters for each drug combination using single-drug responses, equipotent combination data, and drug interaction scores (see Methods); and (3) calculating the killing rate for each combination based on these derived parameters. This approach provides a more comprehensive and scalable prediction of drug interactions than standard partial checkerboard methods (Figure 4).

### Mapping growth conditions to model PD with drug-drug interactions in GranSim by simulating clinically relevant regimens

As our goal in this work is to combine different drug screening approaches to improve regimen screening, in this work we combine *in vitro* combination response data for different bacterial growth conditions into *GranSim* for different drug regimens. In *GranSim*, we assume Mtb can assume three different phenotypes based on their distinct location: intracellular Mtb within macrophages, extracellular-replicating Mtb in granulomatous tissue and nonreplicating Mtb trapped within granuloma caseous-necrotic core. In GranSim, Mtb is modeled as existing in three distinct phenotypic states, reflecting well-characterized spatial niches within the granuloma: intracellular Mtb within macrophages, extracellular replicating Mtb in the surrounding tissue, and nonreplicating Mtb sequestered in the caseous-necrotic core. Using previously published *in vitro* data, we selected three *in vitro* models that together predicted treatment outcomes in the BALB/c relapsing mouse model (Larkins-Ford et al., 2021; Larkins-Ford et al., 2022): these included media with different sole carbon sources (butyrate or 0.2 mM cholesterol) and an acidic condition in standard growth medium. To capture drug pharmacodynamics (PD) relevant to specific Mtb subpopulations, we used the average killing rates from acidic and butyrate conditions to represent intracellular Mtb and the average rates from butyrate and high cholesterol to represent extracellular replicating Mtb. To model the PD of nonreplicating Mtb residing in caseous tissue, we developed a series of *in vitro* dormancy conditions varying in pH and oxygen levels: acidic normoxic (AN), neutral hypoxic (NH), and neutral normoxic (NN) (Methods and Table 1). Drug combinations were evaluated in these dormancy conditions using the DiaMOND methodology (Methods). We evaluated five clinically relevant combinations (HRZE, HRZM, RMZE, BPaL, and BPaMZ) under each of these dormancy conditions to evaluate their ability to predict clinical outcomes.

**TABLE 1:** Characteristics of *in vitro* dormancy conditions (NH, NN, AN) mimicking lesion microenvironments in Mtb infection. Each condition models a distinct set of features that we expect Mtb to encounter within lesions. Together, they are designed to capture the microenvironment heterogeneity that influences drug susceptibility and metabolic state (Sarathy et al., 2018; Sarathy et al., 2019; Wayne and Sohaskey, 2001).

	Neutral-Hypoxic (NH)	Neutral-Normoxic (NN)	Acidic-Normoxic (AN)
Characteristics of each condition	pH: 7, no oxygen exposure, lipid rich	pH: 7, high oxygen exposure, lipid rich	pH: 5.5, high, oxygen, lipid rich
Lesion microenvironment by condition	Necrotic core, center of lesion, poorly vascularized, low oxygen, dense cellular debris	Cavitary lesions, open structure, high oxygen exposure, multiple regions distant from the core	Outer necrotic core, ring of foamy macrophages, high oxygen exposure, transient zone surrounding the core

*In vitro* data measured in different growth stressors have been found to be a better predictor of *in vivo* outcomes than *in vitro* data from a single stressor alone (Larkins-Ford et al., 2021). Based on this prior work, we sought to generate composite stress condition profiles to parameterize GranSim. To do so, we first calculated the killing rate of each drug regimen under the individual stress conditions comprising the combination. We then aggregated these values using either the average, maximum or minimum killing rate, depending on the chosen strategy for modeling the combined effect, which results in the following combined conditions: AN_NH-min, AN_NH-max, AN_NH-mean, AN_NN-min, AN_NN-max, AN_NN-mean, NN_NH-min, NN_NH-max, NN_NH-mean, AN_NN-min, AN_NN-max, AN_NN_mean, AN_NN_NH-min, AN_NN_NH-max, AN_NN_NH-mean. For example, to aggregate all stress conditions in GranSim and to calculate the killing rate of a drug combination against Mtb considering the maximum of all conditions (AN_NN_NH-max), we determine the concentration of drugs an Mtb is exposed to on the simulation grid and calculate the killing rate of that drug combination considering each stress condition (AN, NN and NH) individually. We then take the maximum of killing rates in AN, NN and NH conditions to determine the killing rate of the aggregated condition AN_NN_NH-max (see Methods for details).

**FIGURE 5:**
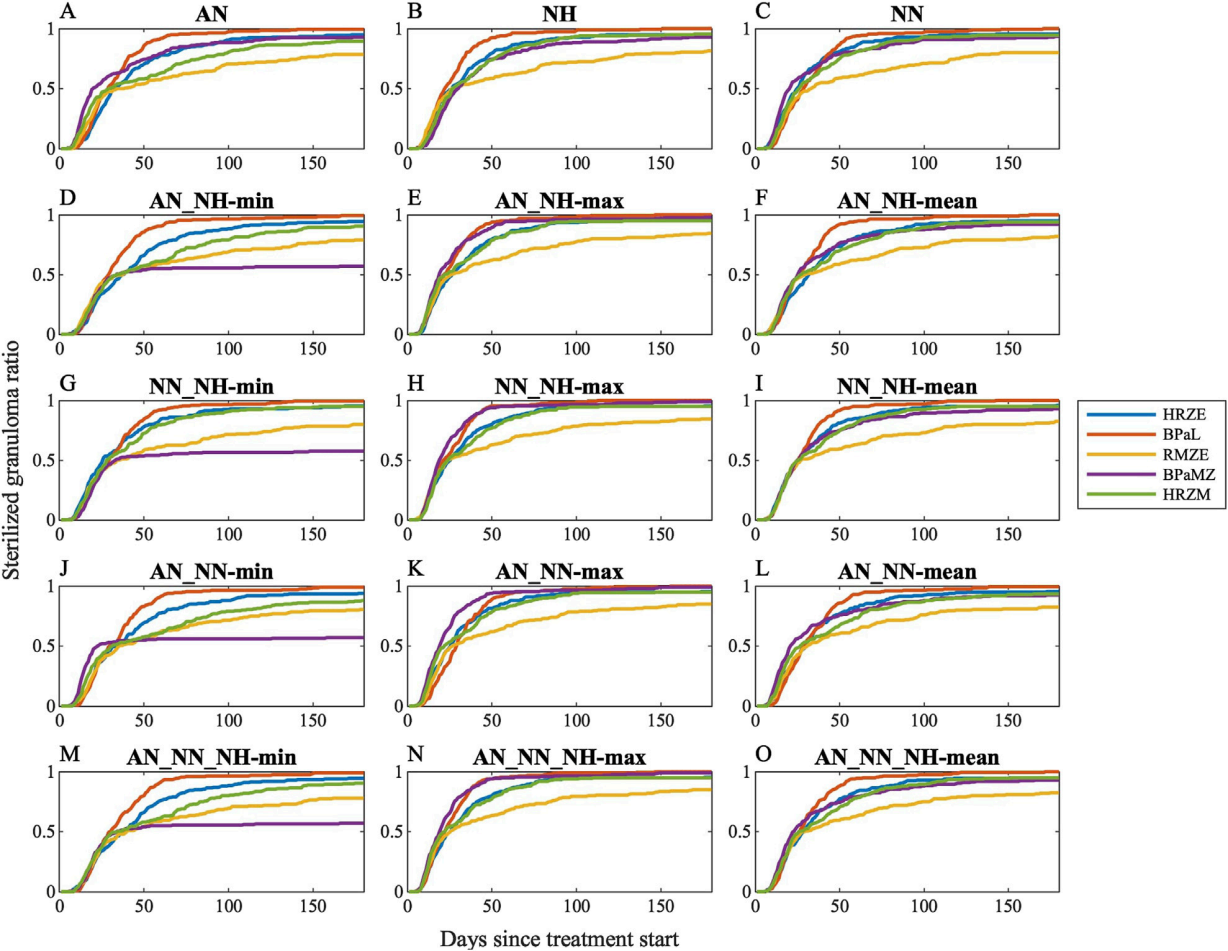
Sterilization curves of five different clinically relevant drug regimens (HRZE, BPaL, RMZE, BPaMZ and HRZM) considering 15 different dormancy spectrum condition combinations: **(A)** acidic normoxic (AN), **(B)** neutral hypoxic (NH), **(C)** neutral normoxic (NN), **(D)** the minimum, **(E)** the maximum, **(F)** the mean killing rate generated by AN and NH, **(G)** the minimum, **(H)** the maximum, **(I)** the mean killing rate generated by NN and NH, **(J)** the minimum, **(K)** the maximum, **(L)** the mean killing rate generated by AN and NN, **(M)** the minimum, **(N)** the maximum, **(O)** the mean killing rate generated by AN, NN and NH. See Supplementary Figure S4 for simulation data.

We first simulated clinically relevant regimens (HRZE, RMZE, HRZM, BPaL and BPaMZ) in GranSim considering all individual conditions and all possible aggregated conditions to determine the condition that is the best representative of clinical or *in vivo* outcomes (Figure 5). As the levels of nonreplicating bacteria trapped within caseum are likely the hardest population to clear with drugs due to drug tolerance and penetration issues, it is not surprising that PD of drugs acting on this population significantly affects sterilization dynamics. For example, BPaMZ has low efficacy assuming nonreplicating Mtb is exposed to certain growth conditions (AN_NH-min, NN_NH-min, AN_NN-min and AN_NN_NH-min, see Figures 5D,G,J,M), whereas BPaMZ sterilizes granulomas fastest when we assume nonreplicating Mtb is exposed to NN_NH-max, AN_NN-max and AN_NN_NH-max (Figures 5H,K,N). Moreover, the assumption that AN represents the microenvironment of nonreplicating Mtb results in a low sterilizing performance of HRZM (Figure 5A). By contrast, HRZM has one of the highest efficacies if one assumes that NN_NH-mean best describes that microenvironment (Figure 5I).

While sterilization dynamics of some regimens significantly depend on the chosen condition, some features of sterilization curves are common amongst most conditions. For example, RMZE performs the worst with almost all tested dormancy models except when BPaMZ has the lowest efficacy as in Figures 5D,G,J,M. In addition, BPaL is generally the best performer except when AN_NN-max is used for the PD of nonreplicating Mtb where BPaMZ sterilizes granulomas fastest (Figure 5K).

**FIGURE 6:**
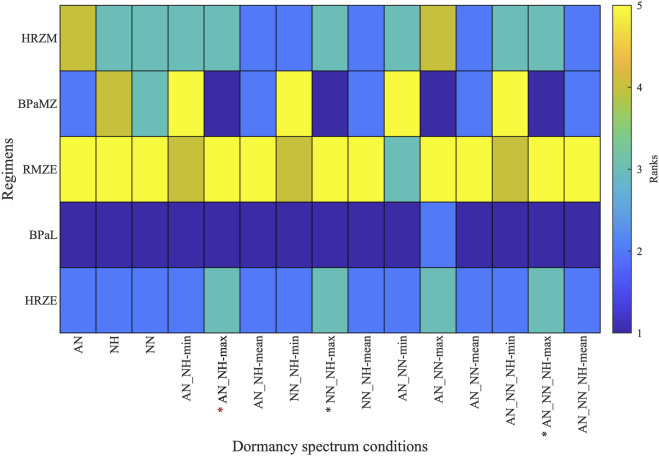
Rankings of five clinically relevant drug regimen behaviors (HRZE, BPaL, RMZE, BPaMZ and HRZM) when considering 15 unique single- and composite dormancy conditions (AN, acidic normoxic; NN, neutral normoxic; NH, neutral hypoxic; min, max and mean correspond to the minimum, maximum or mean of the killing rates generated by the growth condition combinations). Regimens with lower ranks have higher efficacies than regimens with higher ranks. See Table 1 for explanation of conditions. Stars below the columns indicate stress conditions that reflect *in vivo* conditions best according to the ranks of clinically relevant regimens. Red star indicates the stress condition chosen for use in our further analyses.

To determine growth conditions that reflect clinical and animal studies in a systematic way, we ranked the same five regimens for all simulated single- and composite dormancy conditions using Equation 24 (Figure 6). To choose which single- or composite dormancy condition we use to represent *in vivo* conditions, our primary criterion is that BDQ and PTM-containing regimens should outperform HRZE as the inclusion of BDQ and PTM has been previously shown to improve the efficacies of regimens compared to HRZE (Tasneen et al., 2016; Dawson et al., 2015; Cevik et al., 2024; Tasneen et al., 2011; Diacon et al., 2015). Only AN_NH-max, NN_NH-max, AN_NN-max, and AN_NN_NH-max fulfill these criteria, where BPaL and BPaMZ are the best-performing regimens (Figure 6). Next, we anticipate that HRZE should not outperform HRZM. The relative performances of HRZE and HRZM are debatable, as some studies claim HRZM has a higher efficacy than HRZE (Imperial et al., 2018; Nuermberger et al., 2004a; Burman et al., 2006; Conde et al., 2009), while clinical outcomes provide evidence that HRZM is not non-inferior to HRZE (Gillespie et al., 2014). Still, to our knowledge, there are no studies showing that HRZE could perform better than HRZM. These two criteria leave us with three conditions: AN_NH-max, NN_NH-max and AN_NN_NH-max. Since each of these conditions have similar sterilization dynamics, we continue our analyses by choosing one and mapping AN_NH-max to the PD of nonreplicating Mtb.

### Validation of GEODE using NHP and clinical studies

In the sections above, we isolate two new components critical to accurate antibiotic simulation - the effect of bacterial levels and a realistic representation of dormancy in *GranSim*. We now include a third feature: *in vitro* measurements of drug-drug interactions (see Methods) to complete our expanded drug simulation platform: GEODE (Granuloma Environment Optimization of Drug Efficacy). GEODE is our novel, drug regimen efficacy prediction model that combines drug-drug interaction PD with our *GranSim* computational model of PK/PD dynamics within a granuloma. To validate GEODE, we compare our treatment simulations to both NHP and clinical studies. We use datasets from two different types of NHPs: macaques and marmosets. *Cynomolgus macaque* are an accurate model of human Mtb infection, whereas common marmosets almost exclusively develop progressive disease (Via et al., 2013; Flynn et al., 2017). We analyzed three features of simulations: (a) temporal CFU trajectories within all granulomas in response to treatment, (b) rankings of regimens based on granulomas sterilization times, and (c) change in overall granuloma metabolic activity after treatment, which can be simulated and has been experimentally quantified via FDG avidity from PET/CT scans (Budak et al., 2023; Lin et al., 2013; White et al., 2017). For (a–c), data from the NHPs are only available for three of our five clinically relevant treatment regimens, namely, HRZE, RMZE and BPaL. Therefore, our comparisons are only for that.

#### CFU trends of granulomas in response to treatment

**FIGURE 7:**
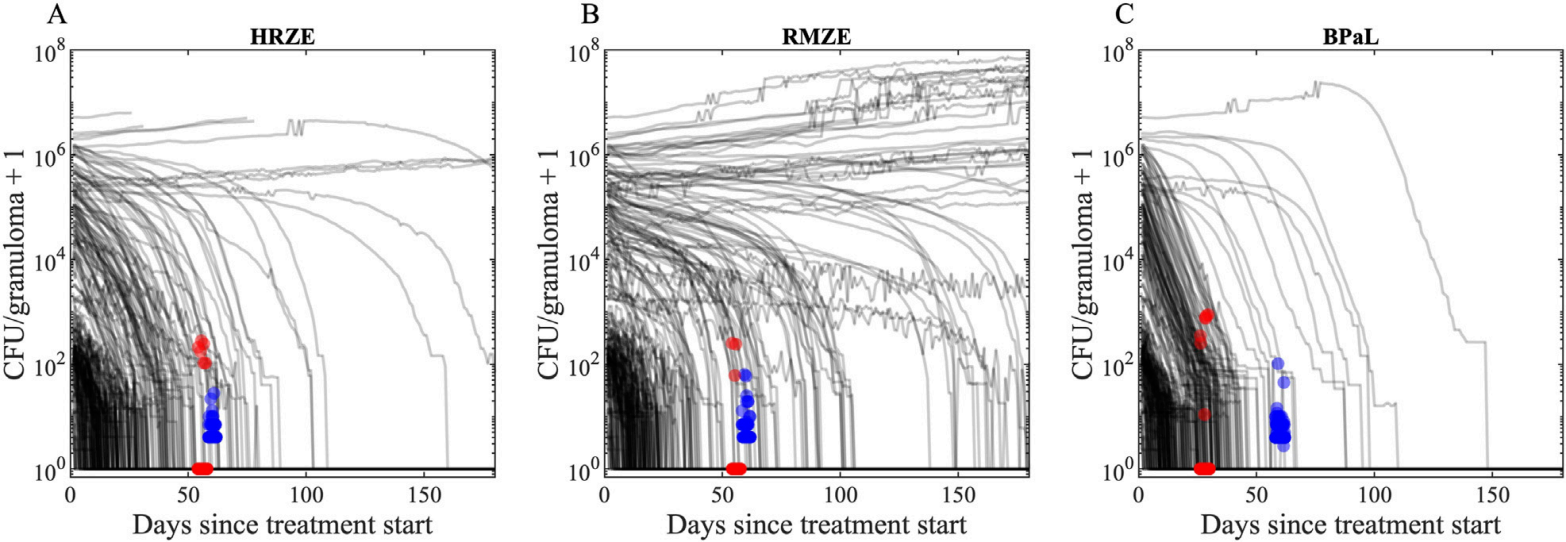
CFU trends of our *in silico* granuloma library with low- and high-CFU granulomas in response to treatment with **(A)** HRZE, **(B)** RMZE and **(C)** BPaL. Black lines indicate simulated CFU trends in response to the corresponding treatment for 180 days. Red dots are data from macaque studies at days 56 for HRZE and RMZE and day 28 for BPaL (Budak et al., 2023; Michael CT et al., 2025), and blue dots are data from marmoset studies at day 60 (Budak et al., 2024). There are **(A)** 51 macaque (45 sterilized) and 40 marmoset (none sterilized) granulomas in HRZE studies, **(B)** 30 macaque (27 sterilized) and 49 marmoset (none sterilized) granulomas in RMZE studies and **(C)** 96 macaque (89 sterilized) and 114 marmoset (none sterilized) granulomas in BPaL studies. To improve visual clarity, a jitter of ± 2 days is added to the x coordinates of marmoset and macaque data. To avoid undefined values of CFUs on the logarithmic y-axis for sterilized granulomas, we added one to the CFU counts.

We first compare CFU trends from our simulated granulomas when treating with three drug regimens (HRZE, RMZE and BPaL) directly to CFU levels from NHP granulomas after administration of either 1 or 2 months of treatment (Budak et al., 2023; Budak et al., 2024) (Figure 7). All treatments in our study result in overall reduced CFU levels in simulated granulomas. In most cases the decline is nonlinear, initially a slow decline followed by a fast decline. Treatments are able to fully sterilize all (e.g., BPaL treatment) or a portion of simulated granulomas (e.g., HRZE or RMZE treatment). We show that our CFU trends are consistent with NHP CFU counts during similar treatment scenarios (Figure 7).

#### Sterilization time-based rankings

**FIGURE 8:**
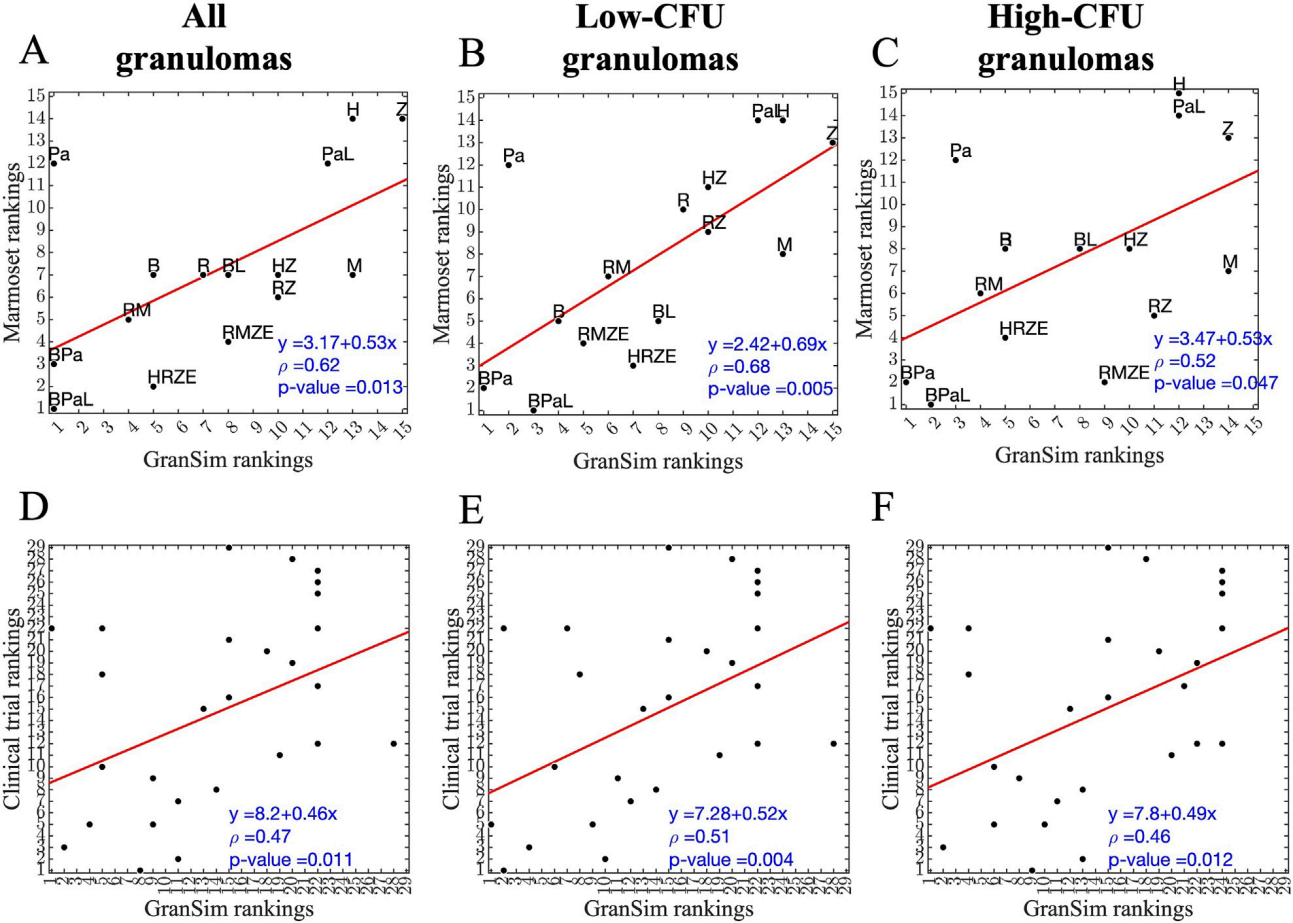
Correlation between simulated and experimental/clinical rankings of regimens based on granuloma-scale sterilization times. **(A–C)** Correlation between marmoset and *GranSim* rankings considering **(A)** all simulated granulomas and all marmoset granulomas, **(B)** simulated low-CFU granulomas and fibrotic marmoset granulomas, and **(C)** simulated high-CFU granulomas and caseous, necrotic marmoset granulomas. **(D–F)** Correlation between simulated and experimental rankings against clinical regimens considering **(D)** all simulated granulomas, **(E)** simulated low-CFU granulomas and **(F)** simulated high-CFU granulomas. In each panel, we calculated correlations using Spearman’s rank correlation, the red line is the best linear fit represented by the equation in the lower right corner, first row of each panel, and ρ is Spearman’s rank correlation coefficient.

To further validate our model, we assess the correlations of rankings between simulations and experimental NHP datasets (Figure 8). We compare GEODE predicted rankings against two *in vivo* rankings: (1) regimen rankings based on marmoset granuloma CFU levels in response to three regimens (*marmoset regimens and rankings*) (Figures 8A–C) (Budak et al., 2024), and (2) *regimen rankings* based on a meta-analysis of Phase 2b clinical trials (*clinical regimens and rankings*) (Figures 8D–F) (Bonnett et al., 2017). To create comparable regimen rankings, we use the GEODE platform to simulate treatments with similar regimens as used in the marmoset regimens and clinical regimens. We rank the simulations based on sterilization times using our ranking method (see Methods) and correlate experimental and simulated rankings as we have done previously (Figure 8) (Budak et al., 2024; Cicchese et al., 2021; Michael CT et al., 2025).

For marmoset regimens, we observe a good correlation between experimental and simulated rankings. Specifically, simulated rankings of low-CFU granulomas correlate well to rankings based on fibrotic marmoset granulomas that tend to have more controlled CFU growth, with a correlation of ρ = 0.68 (Figure 8B). The correlation of high-CFU granuloma rankings to necrotic marmoset granuloma rankings is lower with a correlation of ρ = 0.52 (Figure 8C). Similarly, as in marmoset regimens, low-CFU granulomas correlate better when comparing to the same regimens used in the clinical rankings (Figure 8E). However, overall correlations are lower for clinical regimens than marmoset regimens (see Supplementary Tables S1–S10 for the rankings shown in Figure 8). Overall, we observe that for the majority of high-CFU granulomas, BDQ and PTM-containing regimens are optimal in simulations.

#### Change in metabolic activity after treatment-comparing to FDG levels from PET/CT studies

**FIGURE 9:**
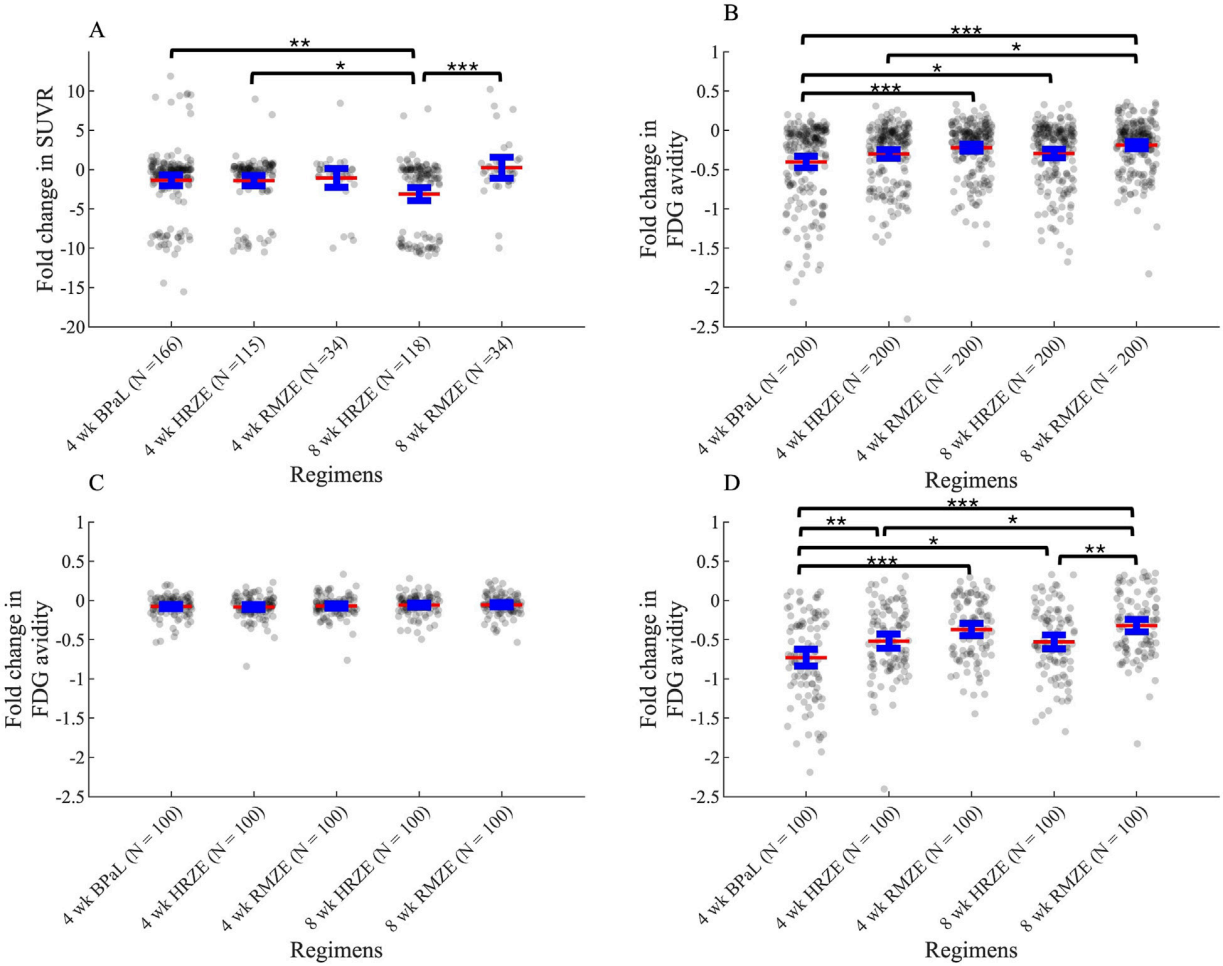
Change in metabolic activity as measured by PET/CT or SUVR in response to three unique treatments in **(A)** macaque studies and in **(B–D)** simulations (see Equations 23, 31). **(A)** Fold change in Standardized Uptake Value Ratio (SUVR) in macaques after treatment compared to SUVR before treatment starts. N indicates the number of total granulomas from 3, two and five macaques treated with HRZE, RMZE and BPaL respectively. **(B–D)** Fold change (log_2_-transformed) in FDG avidity after treatment normalized to the FDG avidity before treatment starts for **(B)** all granulomas, **(C)** only low-CFU granulomas and **(D)** only high-CFU granulomas (*p < 0.05, **p < 0.01, ***p < 0.001, one-way ANOVA with Tukey’s adjusted p values). The central red lines represent the mean, the blue error bars represent the 95% confidence interval.

To evaluate drug efficacies of three unique antibiotic regimens, we also measure metabolic activity of lung granulomas during the course of the treatment. In NHP studies, granulomas are tracked over time within animal and measured for size and FDG avidity. In simulations we also capture this measurement in the form of SUVR (see Methods). We compare the two scores between GEODE simulations and NHP studies. The fold change of SUVR in NHP granulomas indicates that most treatments tend to decrease metabolic activity within granulomas, as simulated mean values of SUVR changes for most treatments (except the mean value of SUVR change after 8 weeks of RMZE treatment) are negative (Figure 9A). However, there are also NHP and virtual granulomas with increased metabolic activity after treatment. We observe that the fold change of SUVR in response to 8 weeks of HRZE treatment is significantly less than that of RMZE, meaning that HRZE reduces the metabolic activity more than RMZE in macaque granulomas. Similarly, at the end of treatment, marmosets treated with RMZE tend to have more severe pathologies than marmosets treated with HRZE and BPaL (Personal communication, C. Barry & B. Aldridge). Marmoset treatment at the granuloma scale with BPaL yields the most improved PET/CT pathology amongst all three regimens (Personal communication, C. Barry & B. Aldridge).

The fold change (log_2_-transformed) in FDG avidity calculated using Equations 22, 23 in all simulated granulomas show similar results as compared to NHP datasets: treatment with these regimens decrease metabolic activity in all cases as shown by negative FDG avidity fold change values (Figure 9B). In addition, RMZE treatment results in the highest FDG avidity change over all three treatments, suggesting that decrease in metabolic activity is lowest in response to RMZE treatment. BPaL treatment shows the largest decrease in metabolic activity. However, there are some differences between the *in silico* and *in vivo* trends in FDG avidity. For example, while the *in vivo* fold change after 8 weeks of HRZE treatment is lower than that observed after 4 weeks, no significant difference is seen between these groups *in silico*. This possibly results from differences in dynamics of metabolic activity; simulations show an exponential decrease in FDG avidity, contrasting with the trends observed *in vivo*. Lastly, changes in FDG avidity between low-versus high-CFU granulomas indicate that differences in FDG avidity amongst regimens results mainly from metabolic differences observed in high-CFU granulomas and those differences are more pronounced in high-CFU granulomas (Figures 9C,D).

## Discussion

Improvement to treatment with TB will be enhanced by better tools to predict efficacies prior to clinical trials. There are a host of tools currently used to this end, but none integrates across platforms to cross from bacterial dynamics to host-scale dynamics. A key goal of this work is to integrate datasets from *in vitro* and *in vivo* studies into our computational model *GranSim* that simulates granuloma formation and function as well as PK/PD dynamics of multiple antibiotic regimens.

In this study, we developed a new computational platform, GEODE, that integrates an *in silico* model of granuloma treatment with *in vitro* PD and PD drug-drug interaction measurements using Mtb grown in various *in vitro* conditions. This model has been calibrated using datasets from NHP models of TB that have been curated for 20 years. This novel methodology is different from our previous studies of *in silico* TB treatment at the granuloma scale (Budak et al., 2023; Budak et al., 2024; Cicchese et al., 2020a), where we used bactericidal (standard media and caseum mimic assays) and macrophage assays to model drug PD and predicted FIC values from INDIGO (Ma et al., 2019) to capture drug-drug interactions. GEODE captures a more nuanced description of TB dynamics in several ways. First, the *in vitro* stress conditions mimic various *in vivo* granuloma microenvironments, so our new PD datasets are more relevant than datasets from standardized assays. Moreover, we measured drug-drug interactions during a variety of stress conditions, whereas previously we used machine-learning based predictions for drug-drug interactions from INDIGO studies (Ma et al., 2019; Cokol et al., 2018). Lastly, PD measurements for all drugs represented in GEODE have been generated using the same experimental platform, making it more consistent than heterogeneously-sourced calibrations.

Our GEODE pipeline involves a detailed methodology to calculate PD of all drug combinations (i.e., the entire checkerboard using the diagonal, x-axis and y-axis) using single-drug PD and equipotent combinations. To our knowledge, this is the first methodology to calculate detailed drug-drug interaction predictions using minimal information. Hence, this approach could minimize resources needed for PD and drug-drug interaction measurements, not only for TB regimens, but for treatments for other diseases requiring checkerboard assays for assessing interactions for combination regimens, such as cancer and HIV (Bayat et al., 2017; Bartlett et al., 2006). An interesting outcome of our calculations is that the contours of the checkerboards may have complex and irregular shapes. This is due to incorporating two different FIC values (FIC50 and FIC90) into our calculations that are not always parallel. This means that the contours can be concave (as in synergistic drug combinations) or convex (as in antagonistic drug combinations) at different parts of the checkerboards (Figure 4C).

An interesting outcome of all stress condition combinations, including the one we chose to use to represent the PD of nonreplicating Mtb within *GranSim* (AN_NH-max), is that regimen RMZE is one of the worst performers within clinically-relevant regimens–even worse than the standard regimen HRZE. The efficacy of RMZE is controversial, as some studies claim that RMZE is better than HRZE (Imperial et al., 2018; Nuermberger et al., 2004a; Nuermberger et al., 2004b; Li et al., 2015; Dorman et al., 2009), while some show the non-inferiority of RMZE to HRZE (Gillespie et al., 2014). However, PET/CT data from marmoset (Personal communication, C. Barry & B. Aldridge) and macaque studies (Figure 9A) demonstrate that granulomas of NHPs treated with RMZE show the least amount of metabolic change. This is in line with changes in FDG avidity of our simulated granulomas, where decreases in levels of FDG avidity in response to RMZE treatment are lowest when compared to both HRZE and BPaL (Figures 9B–D). It is also worth noting that FDG avidity is a less precise indicator of regimen performance than sterilization time, as the differences between regimens are not always significant (e.g., BPaL and HRZE in Figure 9B). Sterilization time provides a clearer measure of treatment efficacy, as it directly reflects the presence or absence of bacteria. In contrast, FDG avidity reflects metabolic activity, which may remain elevated even in the absence of viable bacteria.

To further validate our PD model, we simulate three drug regimens for comparison with both marmoset studies and clinical trials. Our low-CFU virtual granulomas correlate best to experimental and clinical studies. Regimen rankings indicate that stress conditions used to generate PD data mimic low-CFU, less caseous granuloma microenvironments. Furthermore, we observe that our simulated regimen rankings agree more with marmoset regimen rankings than published clinical rankings. This might be due to several reasons. First, clinical rankings occur at a whole host-scale, unlike our granuloma-scale simulations. Clinical rankings would include other extrapulmonary factors such as lymph node infections and disease relapse or the effect of hosts having multiple lung granulomas. Next, regimens from clinical studies are combinations of these five drugs HRZEM only and only dosages and administration frequencies vary. We pooled several different regimens to match their HRZE ranking. Therefore, it may be harder to reflect those subtle differences between simulations and clinical studies, leading to weakly correlated rankings. However, regimens from marmoset studies have a wider variety of PKPD features with fixed dosages and frequencies, making it easier to distinguish rankings. Our high correlations between simulations and NHP data indicate that we were able to successfully capture the PKPD dynamics of all regimens under clinical daily dosing conditions.

While correlation coefficients are informative to evaluate models, they do not capture systematic biases in predictions. The linear fits of our correlation plots indicate that our model slightly underpredicts experimental and clinical rankings. This may be due to the lack of host-scale dynamics, like lymph nodes or multiple granulomas. To further improve our clinical predictions, current work has focused on adapting this methodology using our whole-host scale model that we developed, namely, HostSim, which includes dynamics of lymph nodes and multiplelung granulomas. We test drug regimens within virtual cohorts generated with this model (Joslyn et al., 2022; Michael et al., 2024). Through this approach, we aim to make more clinically-relevant predictions.

We observe some outliers in our correlation plots for both clinical and marmoset analyses. The striking difference between marmoset and *GranSim* is the rankings of PTM, which performs significantly better in *GranSim*. This may be due to marmosets developing more progressive disease faster, with more caseous granulomas preventing effective penetration of PTM, significantly affecting its efficacy. In correlation plots between *GranSim* and clinical rankings, regimens with solely INH or RIF tend to perform better in *GranSim* than as compared to clinical trials. RIF can penetrate well into granulomas, hence it performs better in our granuloma-scale model. However, in the clinic, we need to use other drugs in combination regimens, such as EMB and PZA, to prevent resistance, which is not currently captured in these simulations.

The effect of bacterial levels is a phenomenon where drug efficacy is decreased due to presence of higher number of microorganisms (inoculum effect in *in vitro* studies) (Brook, 1989). This phenomenon has previously been observed with several bacterial species, such as *E coli, S aureus, K pneumoniae,* and Mtb (Brook, 1989; Kirby and Dubos, 1947). A mathematical model has been previously developed to characterize effects of bacterial burden using ODEs, where the effective concentration of drugs decreases with increasing bacterial population (Bhagunde et al., 2010). Here, we incorporate effects of bacterial burden into GEODE similarly by modifying drug killing rates based on the number of bacteria in granulomas. We calibrate this using *in vitro* data that show PD of different antibiotics when administered to bacterial plates with different bacterial loads. As expected, we observe a significant reduction in granuloma sterilization rates due to effects of bacterial levels (Figure 3) consistent with *in vivo* measurements. We noticed that ratios of sterilized granulomas from macaques are comparable with sterilized granuloma ratios of simulated low-CFU granulomas including bacterial burden dynamics. By contrast, sterilized granuloma ratios of marmosets are comparable with sterilized granuloma ratios of simulated high-CFU granulomas, when we include bacterial burden effects.

We are able to match outcomes of low- and high-CFU granulomas (as in the results for our bacterial burden model or post-infection CFU trends) to macaque and marmoset granulomas, respectively. This follows as the majority of macaque granulomas have controlled CFU growth, like our simulated low-CFU granulomas (Maiello et al., 2018; Flynn et al., 2017), and marmoset granulomas tend to have high, uncontrolled CFU growth like our simulated high-CFU granulomas (Via et al., 2013; Flynn et al., 2017). However, our analyses on metabolic activity of granulomas in response to treatment indicate that high metabolic activity of RMZE is observed in each of macaque, marmoset and simulated high-CFU granulomas, respectively. This is counterintuitive; however, several studies showed that FDG avidity is not correlated to CFU levels (Lin et al., 2013). Another counterintuitive result is that fibrotic marmoset rankings correlate better to low-CFU granulomas, as evidenced by a higher correlation coefficient ρ. This is likely due to our granuloma stress conditions representing microenvironments within cellular granulomas, which tend to control CFU growth, similar to our simulated low-CFU granuloma set.

GEODE is a novel and powerful tool to assess TB regimen efficacies and to calculate PD and drug-drug interactions for higher order combinations (combinations with three or more drugs), which requires data from equipotent combinations. This means that we need to generate equipotent data for all combinations of interest. However, the methodology can be improved so that PD of higher order combinations can be predicted from all 2-way combinations. For example, this could be done by integrating a dose-response model into our approach. This is a mathematical model predicting efficacy of higher order combinations using underlying drug-drug pairs (Katzir et al., 2019). This would significantly reduce the required number of experiments for our GEODE pipeline and would allow us to test all possible higher order combinations without additional experiments.

An additional area that can be improved is that the current version of GEODE primarily predicts the outcome of granulomas with controlled CFU burden, i.e., low-CFU granulomas. However, high-CFU granulomas with uncontrolled CFU growth are harder-to-treat granulomas that lead to treatment failure. Optimizing treatment for high-CFU granulomas would make GEODE a more powerful tool to improve TB treatments. To do that, new stress conditions that mimic caseous, high-CFU granuloma microenvironments can be designed to generate PD data for *GranSim*. This version of GEODE would potentially predict treatment outcomes in high-CFU granulomas, as the CFU trend of caseous granulomas tend to increase uncontrollably. We aim to address these issues in future work to improve GEODE.

## Methods

To create the new pipeline GEODE, it is necessary to perform *in vitro* studies to determine drug dynamics, update each existing model component of *GranSim*, then link them. We describe each step of the GEODE pipeline (Figure 1).

### *GranSim* model of pulmonary granulomas

*GranSim* is a computational, hybrid, multi-scale, agent-based model that we have developed and continuously curated with NHP data for 2 decades; *GranSim* simulates host-pathogen interactions upon Mtb infection in an area of lung tissue (6 mm × 6 mm) (Segovia-Juarez et al., 2004; Ray et al., 2009; Fallahi-Sichani et al., 2011; Cilfone et al., 2013). *GranSim* contains cellular agents, such as immune cells (e.g., macrophages, T cells), Mtb and signaling molecules (chemokines and cytokines). We define the rules that describe the cellular and molecular processes and the agent interactions within *GranSim* based on experimental observations from NHP studies (for a complete description of our rules, see http://malthus.micro.med.umich.edu/GranSim/). We represent these rules using mathematical equations containing parameters and we calibrated all parameters to NHP datasets (Segovia-Juarez et al., 2004; Ray et al., 2009; Cilfone et al., 2013). Each simulation begins with a single infected macrophage in the center of the lung tissue grid that triggers recruitment of immune cells to the infection site. In simulations, structured granulomas are a consistent emergent result of the Mtb and immune dynamics, and that structure shares similar spatial features as NHP and human granulomas—e.g., a necrotic core, a cellular rim composed of macrophages surrounding the necrotic core, and a T-cell cuff as the outermost ring.

To capture the heterogeneity of Mtb metabolic states, we included three types of Mtb based on their location within a granuloma: intracellular Mtb reside within macrophages, nonreplicating Mtb are those trapped in the necrotic core, and replicating extracellular Mtb are those within granulomatous tissue (i.e., the non-necrotic region). These Mtb phenotypes have distinct features in terms of replication rates, interactions with immune cells and susceptibility to anti-TB drugs (Lenaerts et al., 2015).

### *In silico* granuloma library

**FIGURE 10:**
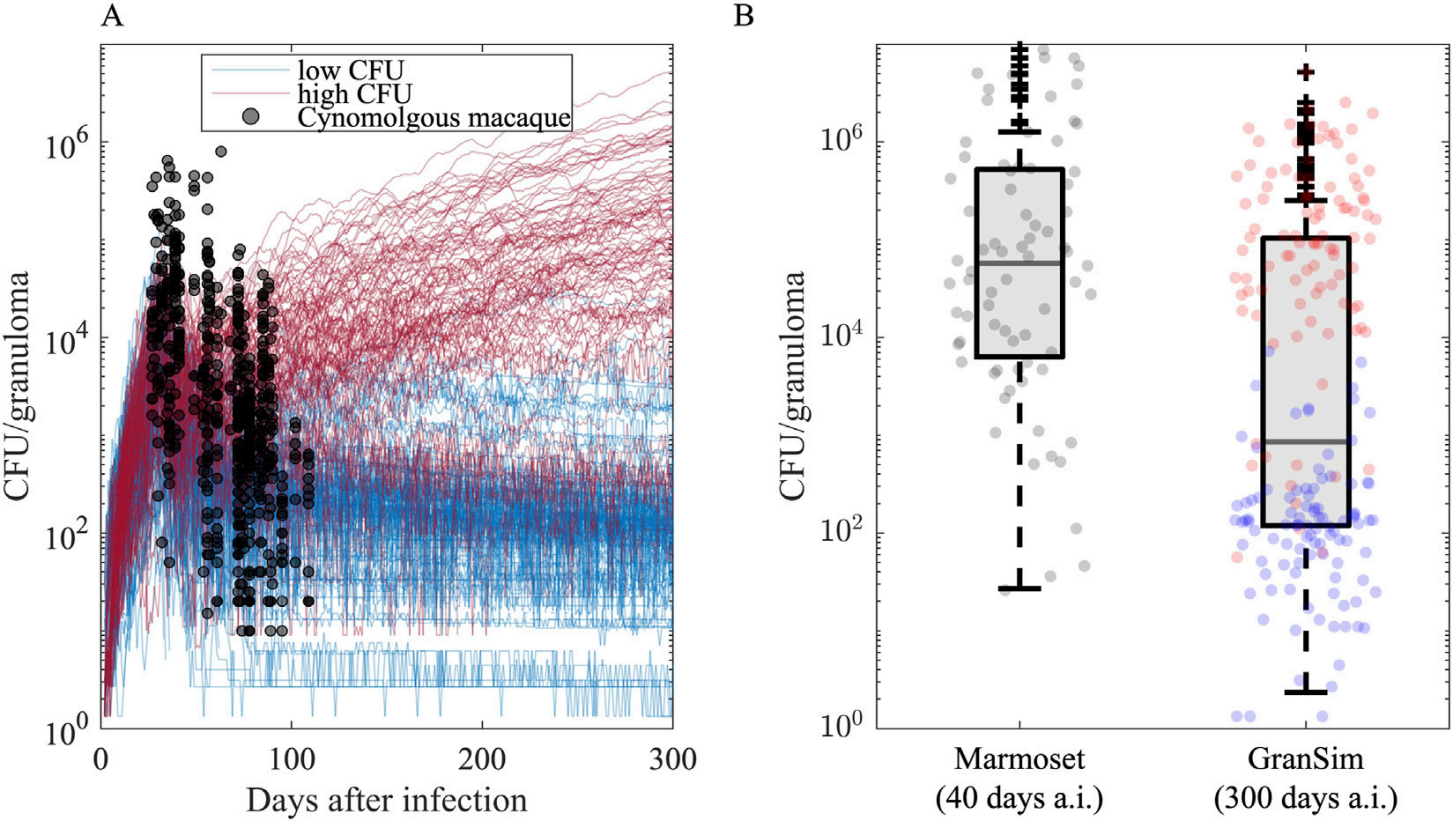
Granuloma CFU trends simulated in *GranSim* following the onset of infection agrees with the data from nonhuman primate (NHP) studies. **(A)** Each curve illustrates the simulation of a single granuloma using a specific parameter set. Black dots are CFU counts of 646 cynomolgus macaque granulomas from 42 untreated *Cynomologus macaques*, where each NHP has 2–40 granulomas (the median is 14.5, 25th and 75th percentiles are 9 and 20, respectively.) (Budak et al., 2023; Joslyn et al., 2022; Hult et al., 2021). We categorize simulated granulomas based on their CFU trends into low-CFU (blue curves, N = 100) and high-CFU (red curves, N = 100) granulomas that represent granulomas with controlled and uncontrolled CFU burden, respectively. **(B)** Comparison of CFU counts of *GranSim* granulomas 300 days after infection to 76 Marmoset granulomas from four untreated marmosets 40 days after infection, where each monkey has 13, 7, 18 and 38 granulomas (Budak et al., 2024). Blue and red dots are from low- and high-CFU virtual granulomas, respectively.

To analyze regimen efficacies at the granuloma scale, we use *GranSim* to generate an *in silico* granuloma library that contains 200 granulomas. We generate this granuloma library by sampling 250 parameter sets within biologically relevant ranges using Latin Hypercube Sampling and simulate granulomas parameterized by each parameter sample for 300 days post-infection including three replications of each (to address aleatory (stochastic) uncertainty (Marino et al., 2008), 750 simulations in total. The parameter ranges are calibrated to CFU counts from macaque granulomas (Figure 10A). We then categorize granulomas into low- and high-CFU granulomas (as described in the next paragraph) and randomly pick 100 low- and 100 high-CFU granulomas from the set of 750 granulomas, having 200 granulomas in total. Due to a random selection process, it is possible to have granulomas with the same parameter set but different stochastic processes (i.e., different seeds that determine stochastic events) within the *in silico* granuloma library.

To assess how effective regimens are in treating different types of granulomas, we grouped them by their capacity to control Mtb growth. To do this, we grouped virtual granulomas into two groups based on their bacterial growth trends after day 300 post-infection: low-CFU and high-CFU granulomas. LTBI patients mainly have sterile or low-CFU granulomas with stable CFU levels, whereas active TB patients likely have at least one high-CFU granuloma with uncontrolled CFU growth (Cicchese et al., 2020a; Lin and Flynn, 2018; Gideon et al., 2015). As in our previous work (Budak et al., 2023; Budak et al., 2024), we define granulomas as *low-CFU* if they have nonzero and less than 10^4^ CFU at the end of 300 days post-infection, and their CFU levels do not increase more than 50 CFU in the last 20 days of simulation (blue curves in Figure 10). We define granulomas as if they have CFUs between 10^4^ and 10^7^ at the end of the 300-day simulation or their CFUs increase more than 50 CFU in the last 20 days of simulation (red curves in Figure 10) (Our model is flexible and can easily update these ranges). Out of 750 simulations, we randomly selected 100 low-CFU and 100 high-CFU granulomas, generating a library of 200 granulomas. Note that marmosets are very small animals and *GranSim* timing was based mostly on macaques and human datasets; thus, infection-dynamic timing in marmosets is different than simulations. In fact, CFU counts from marmoset granulomas correlate better with later time points in infection dynamics as simulated by *GranSim* (Figure 10B).

### Pharmacokinetic (PK) model in *GranSim*

We previously included a PK model within *GranSim* that simulates spatial distributions of antibiotics within simulated granulomas following administration of antibiotics (Pienaar et al., 2015a; Pienaar et al., 2017; Pienaar et al., 2015b), which we continue to use in the present work. Briefly, we first simulate plasma PK using a compartmental model with a system of ordinary differential equations. Drugs concentrations in plasma permeate into lungs, which we model via flux terms of drugs from plasma onto the simulation lung grid through vascular source agents. Once a drug permeates into simulated lung tissue, it diffuses through cellular tissue, binds to macromolecules (e.g., caseum, epithelial tissue, etc.), and partitions into macrophages (see http://malthus.micro.med.umich.edu/GranSim/ for the full list of equations and code files). We calibrate the parameters that define PK processes using *in vivo* studies from humans or animal models (Cicchese et al., 2020a; Budak et al., 2023; Budak et al., 2024). *GranSim* currently represents eight antibiotic drugs used for treatment of TB: isoniazid (H or INH), rifampicin (R or RIF), pyrazinamide (Z or PZA), ethambutol (E or EMB), moxifloxacin (M or MXF), Bedaquiline (B or BDQ), Pretomanid (Pa or PTM), Linezolid (L or LZD).

### *In vitro* assays

*In vitro* assays were performed using DiaMOND experimental design in *Mycobacterium tuberculosis* (Erdman strain). *In vitro* data for the cholesterol, butyrate, and acidic conditions were obtained from Larkins-Ford et al. (2021). For the dormancy (non-replicating) conditions in this study (Table 1), *M. tuberculosis* was initially cultured in Middlebrook 7H9 broth supplemented with OADC and Tween-80, then acclimated to a lipid-rich medium containing oleic acid, palmitic acid, arachidonic acid, linoleic acid, stearic acid, and cholesterol.

The dormancy media were buffered to a pH of 5.5 for acidic conditions and pH 7.0 for neutral conditions using MES (2-(N-morpholino)ethanesulfonic acid) and MOPS (3-(N-morpholino)propanesulfonic acid), respectively. Normoxic cultures were maintained under ambient oxygen (vented flasks), while hypoxic cultures were incubated in low-oxygen conditions (non-vented flasks). The dormancy cultures were incubated at 37 °C for 4 weeks prior to drug treatment, followed by 1 week of drug exposure. Given the low metabolic state of non-replicating cells, cultures were plated on charcoal agar (Gold et al., 2016) to inactivate residual antibiotics and promote outgrowth. Luminescence was measured to assess bacterial survival, as the Mtb strain used in this study carries the autoluminescent reporter plasmid pMV306hsp+LuxG13 (Larkins-Ford et al., 2021). All assays were performed in biological triplicate, and the median value across replicates was used for downstream analyses (see Supplementary File 2 for the raw data).

### Checkerboard calculation method

We create a methodology for predicting bactericidal activities of various drug dose combinations. Note that we distinguish between two major measurements of the potency of a drug: bactericidal activity and bacterial inhibition. To infer the bactericidal activity of multi-drug combinations in GEODE, we use data provided from *in vitro* assays (Cokol et al., 2017; Larkins-Ford et al., 2021) in the form of fractional inhibitory concentration values (FIC, see Glossary). Concretely, inhibitory results are represented by a Hill function that defines the level of bacterial growth inhibition as a function of drug concentration. The general form of a Hill curve can be represented as

$$E\left(C\right)=\text{Emax}\frac{C^{h}}{C^{h}+{C50}^{h}}$$ (1)

where E is the effect of a drug that depends on its concentration C , Emax is the maximum effect a drug can reach, C50 is the concentration needed to reach the half-maximum effect ( Emax /2) and h is the Hill constant, which characterizes the steepness at the inflection point. Here, we present two sets of Hill curve parameters: growth inhibition-based parameters ( ${\text{Emax}}_{inh}$ ; $C50_{\text{inh}}$ , $h_{\text{inh}}$ ) obtained from *in vitro* assays and, inferred from that in the next subsection, bactericidal activity-based parameters ( ${\text{Emax}}_{\left\{\text{frac}\_\text{killed},i\right\}},{\text{Emax}}_{i}$ , ${C50}_{\left\{N,i\right\}}$ , $h_{i}$ for drug i ; ${\text{Emax}}_{comb}$ and ${\text{Emax}}_{\left\{\text{frac}\_\text{killed},\text{comb}\right\}}$ for combinations).

Datasets from these assays represent growth inhibition-based PD for single-drug dynamics (x- and y-axis of a standard checkerboard assays) and equipotent drug combinations (diagonals of the same checkerboard) (Larkins-Ford et al., 2021). Herein we derive a method to calculate the bactericidal activities of all possible dose combinations (i.e., the whole checkerboard) by using growth-inhibition-based dose response curves for single drugs and the equipotent combinations, and colony counts for different growth inhibition levels.

#### Conversion of *in vitro* growth inhibition-based parameters to bactericidal activity-based parameters

##### Conversion of Hill curve parameters

**FIGURE 11:**
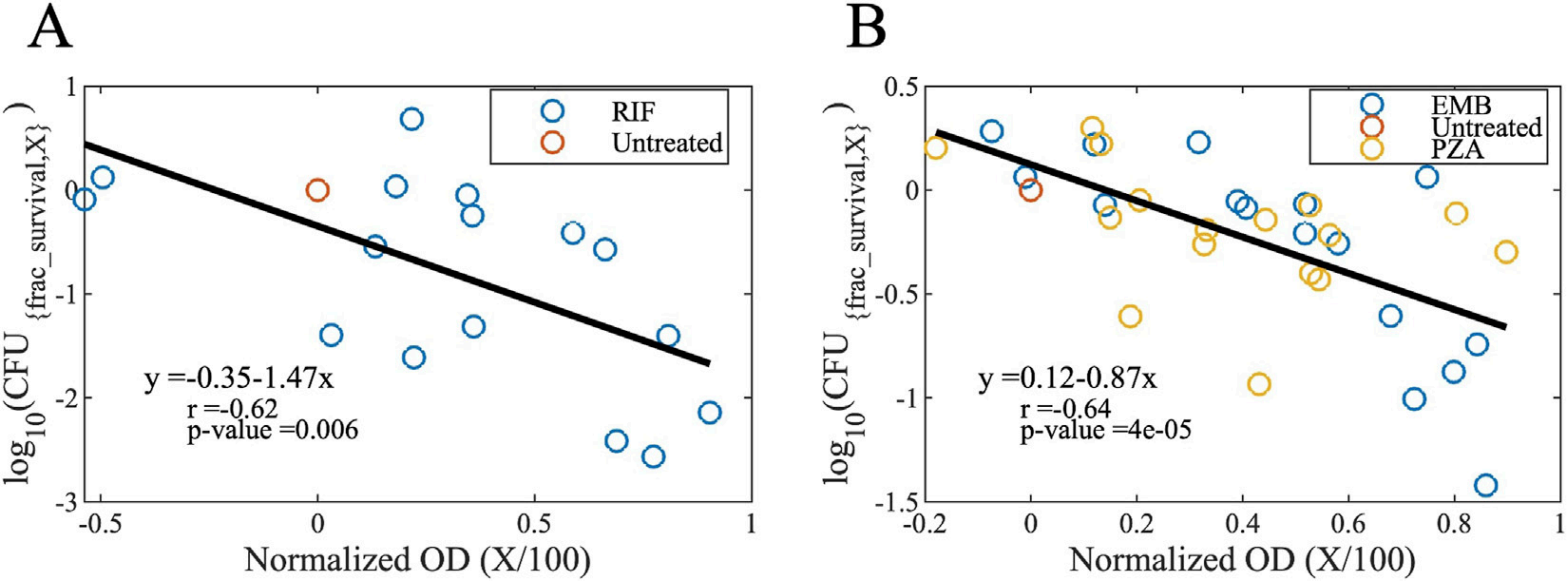
Maps to convert growth inhibition levels to bactericidal activity levels for **(A)** bactericidal and **(B)** bacteriostatic drugs (see Equation 2). Normalized OD values (OD_600_ values normalized to the untreated case) correspond to the growth inhibition level ratios (growth inhibition level X divided by 100). For example, a normalized OD value of 0.5 means a 50% of growth inhibition which is caused by a drug concentration of IC_50_. The y-axis refers to the log_10_ fraction of survived CFU levels; a value of −1 means 10% of the bacteria is survived and 90% died by exposure to a drug concentration of BC_90_ (OD, optical density; CFU, colony forming unit; X, growth inhibition level; RIF, rifampicin; EMB, ethambutol; PZA, pyrazinamide).

As the PD model in *GranSim* for drug action is based on bactericidal activity of drugs, we convert the growth inhibition-based Hill curve parameters ( ${\text{Emax}}_{inh}$ ; $C50_{\text{inh}}$ , $h_{\text{inh}}$ ) and inhibitory concentrations (ICs) to bactericidal activity-based Hill curve parameters and bactericidal concentrations (BCs). To do this, we generate maps that determine relative CFU corresponding to the optical densities of bacterial cultures (OD_600_), a measure proportional to the concentration of bacteria, for bactericidal (Figure 11A) and bacteriostatic drugs (Figure 11B). The logarithm of the normalized CFU counts (CFU counts normalized to the untreated case, i.e., fractional survival) decreases linearly for increasing normalized OD values (OD_600_ values normalized to the untreated case), also known as growth inhibition level (X/100, see IC_X_ in Glossary):

$$\log_{10}\left({\text{CFU}}_{\left\{\text{frac}\_\text{survival},X\right\}}\right)=m\cdot X/100+n$$ (2)

where X is the growth inhibition level, ${\text{CFU}}_{\left\{\text{frac}\_\text{survival},X\right\}}$ is the fraction of live bacteria after being treated with ${\text{IC}}_{X}$ , m and n are the constants for the best linear fit ( m=−1.47 and n=−0.35 for bactericidal drugs, m=−0.87 and n=0.12 for bacteriostatic drugs).

We used these linear maps (see Equation 2) to calculate fractional survivals of bacteria when they are exposed to various drug concentrations. Those drug concentrations are IC values (IC_10_, IC_25_, IC_50_, IC_75_, IC_90_ and IC_95_, see Glossary) determined by the OD_600_ values of drug-treated bacterial cultures, which is used to derive growth inhibition-based Hill curve parameters for single drugs and equipotent combinations. We fit these concentration-to-fractional survival maps to a Hill curve to obtain bactericidal activity-based hill curve parameters ( Emax , C50 and h , see Equation 1) for both single drugs and equipotent combinations to be used in *GranSim*. To find the best fits for these parameters, we use

$$\frac{\text{dB}}{\text{dt}}=-k\cdot B$$ (3)

where B is the bacterial level (i.e., CFU counts), and k is the drug killing rate defined as

$$k\left(C\right)=\text{Emax}\frac{C^{h}}{C^{h}+{C50}^{h}}$$ (4)

Additionally, we calculate the maximal fraction of killed bacteria ( ${\text{Emax}}_{\left\{\text{frac}\_\text{killed}\right\}}$ ) using the linear maps and ${\text{Emax}}_{inh}$ as:

$${\text{Emax}}_{\left\{\text{frac}\_\text{killed}\right\}}=1-10^{m\cdot {\text{Emax}}_{inh}+n}$$ (5)

For calculations below, we refer to these calibrated parameters as ${\text{Emax}}_{\left\{\text{frac}\_\text{killed},A\right\}},{\text{Emax}}_{A}$ , ${C50}_{\left\{N,A\right\}}$ ( $C50_{A}$ normalized to ${\text{BC}90}_{A}$ ) and $h_{A}$ for the parameters of drug A calibrated using Equations 1–5. We refer to Emax of bactericidal activity of the equipotent combination as ${\text{Emax}}_{\text{comb}}$ and maximal fraction of killed bacteria using equipotent combination as ${\text{Emax}}_{\left\{\text{frac}\_\text{killed},\text{comb}\right\}}$ .

##### Conversion of FIC values to fractional bactericidal concentration (FBC) values

Using the linear maps (Figure 11), we convert growth inhibition levels to bactericidal killing levels. For example, if a drug concentration inhibits X% of bacterial growth and kills Y% of bacteria, then IC_X_ = BC_Y_, which means FIC_X_ = FBC_Y_. With this method we generate FBC_50_ and FBC_90_ of the equipotent combination. During the calculations below, we will refer to FBC_50_ and FBC_90_ of the equipotent combination as ${\text{FBC}50}_{\text{Diag}}$ and ${\text{FBC}90}_{\text{Diag}}$ , respectively.

#### Derivation of Hill curve parameters based on drug concentrations within a combination

Initially, we derive the bactericidal activity-based Hill curve parameters of all single drugs and equipotent drug combinations (i.e., diagonals on the checkerboard) in **A**. Using these derivations, we determine the parameters for all off-diagonal combinations. We normalize the bactericidal concentrations for each drug to compute fractional bactericidal concentrations by assuming a linear transition between the $E_{\max}$ of an individual drug and a two-way combination of drugs. This allows us to know the effective killing rate of an arbitrary combination of drugs (i.e., a completed checkerboard) given a normalized concentration of each drug. We estimate these effective concentrations by leveraging the Bliss Independence Model (Bliss, 1939) to compute overall kill rate. As an example, we walk through the steps below for the case with two drug combinations, drug A and drug B with concentrations C_A_ and C_B_.

i. *Derivation of* ${Emax}_{\left\{comb,obs\right\}}$

  We assume that Emax changes linearly between the ${\text{Emax}}_{comb}$ and individual Emax values ( ${\text{Emax}}_{A}$ and ${\text{Emax}}_{B}$ , whichever is in excess) with the ratio of concentrations of A and B normalized to their BC90 values ( ${\text{Conc}}_{\left\{N,A\right\}}$ and ${\text{Conc}}_{\left\{N,B\right\}}$ ). This assumption is justified as follows: The drug with the lower dose units is paired with the other drug to behave as the combination ( ${\text{Emax}}_{comb}$ ) and the remainder of the drug in excess behaves as a single drug. We then take the weighted average of Emax values, such that the Emax of combination of interest varies between ${\text{Emax}}_{comb}$ and ${\text{Emax}}_{A}$ or ${\text{Emax}}_{B}$ , whichever is in excess (Supplementary Figures S1A–C).

$${\text{Emax}}_{\left\{\text{comb},\text{obs}\right\}}={\text{Emax}}_{i}+\left(\frac{{\text{Conc}}_{\left\{N,j\right\}}}{{\text{Conc}}_{\left\{N,i\right\}}}\right)\left({\text{Emax}}_{comb}-{\text{Emax}}_{i}\right)\text{ for }{\text{Conc}}_{\left\{N,i\right\}}>{\text{Conc}}_{\left\{N,j\right\}}$$ (6)

For example, if ${\text{Emax}}_{A}$ is 0.8 and ${\text{Emax}}_{comb}$ is 0.9, the combination of 0.1 B ( ${\text{Conc}}_{\left\{N,B\right\}}$ ) and 0.2 A ( ${\text{Conc}}_{\left\{N,A\right\}}$ ) would have an Emax ( ${\text{Emax}}_{\left\{\text{comb},\text{obs}\right\}}$ ) as 0.85 by Equation 6.

ii. Derivation of ${BC50}_{obs}$ and ${BC90}_{obs}$

We first derive ${\text{BC}50}_{\exp}$ and ${\text{BC}90}_{\exp}$ (BC50 and BC90 values of the combination of interest under the assumption that there is no drug-drug interaction involved) by taking the weighted average of the BC values that are normalized to their BC90 values ( ${\text{BC}50}_{\left\{N,A\right\}}$ and ${\text{BC}90}_{\left\{N,A\right\}}$ for drug A, ${\text{BC}50}_{\left\{N,B\right\}}$ and ${\text{BC}90}_{\left\{N,B\right\}}$ for drug B), assuming that both drugs contribute equally to the BC values of the combination (Supplementary Figure S2):

$${\text{BC}\left\{i\right\}}_{\exp}=\frac{{\text{Conc}}_{\left\{N,A\right\}}\cdot {\text{BC}\left\{i\right\}}_{\left\{N,A\right\}}+{\text{Conc}}_{\left\{N,B\right\}}\cdot {\text{BC}\left\{i\right\}}_{\left\{N,B\right\}}}{{\text{Conc}}_{\left\{N,A\right\}}+{\text{Conc}}_{\left\{N,B\right\}}}$$ (7)

where i=50 or 90 .

To derive ${BC50}_{obs}$ and ${BC90}_{obs}$ of the combination of interest, we use FBC values (see Equation 5 in Glossary). We first derive ${\text{FBC}50}_{obs}$ and ${\text{FBC}90}_{obs}$ , the FBC50 and FBC90 of the combination of interest. Here, we assume FBC values of different angles of the checkerboard (i.e., different relative concentrations of drugs–for example, the diagonal of 45° is the equipotent combination, the x- and y-axes correspond to 0 and 90° with drug 1 or drug 2 only, respectively) vary quadratically with the angle between 1 and FBC values of the diagonal, which results in the expected concave and convex contours for synergistic and antagonistic drug interactions, respectively (Supplementary Figures S1D–F):

$${\text{FBC}\left\{i\right\}}_{obs}={\text{FBC}\left\{i\right\}}_{Diag}+{{\text{Conc}}_{\text{diff}}}^{2}\left(1-{\text{FBC}\left\{i\right\}}_{Diag}\right)$$ (8)

for i=50 or 90 .

Here, $\text{Con}c_{\text{diff}}$ is the normalized concentration difference between drugs A and B, which varies between 0 (at the diagonal) and 1 (single drug cases at the x- and y-axis), calculated as (Supplementary Figure S3)

$${\text{Conc}}_{\text{diff}}=\frac{\left|{\text{Conc}}_{\left\{N,A\right\}}-{\text{Conc}}_{\left\{N,B\right\}}\right|}{{\text{Conc}}_{\left\{N,A\right\}}+{\text{Conc}}_{\left\{N,B\right\}}}$$ (9)

We then calculate ${BC50}_{obs}$ and ${BC90}_{obs}$ as:

$${\text{BC}\left\{i\right\}}_{obs}={\text{BC}\left\{i\right\}}_{\exp}\cdot {\text{FBC}\left\{i\right\}}_{obs}\;\text{where}\;i=50\text{or}90.$$ (10)

iii. Derivation of $h_{\left\{comb,obs\right\}}$ and ${C50}_{\left\{N,comb,obs\right\}}$

Using ${\text{BC}50}_{\text{obs}}$ , ${\text{BC}90}_{\text{obs}}$ calculated with Equations 7–10 and ${\text{Emax}}_{\text{frac}\_\text{killed}}$ , we infer $h_{\left\{comb,obs\right\}}$ and ${C50}_{\left\{N,comb,obs\right\}}$ by assuming that the concentrations ${\text{BC}90}_{\text{obs}}$ and ${\text{BC}50}_{\text{obs}}$ lead to 90% and 50% bacterial killing, respectively:

$$0.9={\text{Emax}}_{\text{frac}\_\text{killed}}\frac{{{\text{BC}90}_{\text{obs}}}^{h_{\left\{comb,obs\right\}}}}{{{\text{BC}90}_{\text{obs}}}^{h_{\left\{comb,obs\right\}}}+{{C50}_{\left\{N,comb,obs\right\}}}^{h_{\left\{comb,obs\right\}}}}$$ (11)

and

$$0.5={\text{Emax}}_{\text{frac}\_\text{killed}}\frac{{{\text{BC}50}_{\text{obs}}}^{h_{\left\{comb,obs\right\}}}}{{{\text{BC}50}_{\text{obs}}}^{h_{\left\{comb,obs\right\}}}+{{C50}_{\left\{N,comb,obs\right\}}}^{h_{\left\{comb,obs\right\}}}}$$ (12)

By solving Equations 11, 12 for $h_{\left\{comb,obs\right\}}$ and ${C50}_{\left\{N,comb,obs\right\}}$ we get Equations 13, 14:

$$h_{\left\{comb,obs\right\}}=\frac{\log\left(\frac{2{\text{Emax}}_{\text{frac}\_\text{killed}}-1}{1.1{\text{Emax}}_{\text{frac}\_\text{killed}}-1}\right)}{\log\left(\frac{{\text{BC}90}_{\text{obs}}}{{\text{BC}50}_{\text{obs}}}\right)}$$ (13)

and

$${C50}_{\left\{N,comb,obs\right\}}={\text{BC}50}_{\text{obs}}{\left(2{\text{Emax}}_{\text{frac}\_\text{killed}}-1\right)}^{1/h_{\left\{comb,obs\right\}}}$$ (14)

iv. Calculating the effective concentration ( ${Conc}_{eff}$ ) assuming Bliss independence model

To calculate the killing rate using Hill curve parameters, we need to determine the effective normalized concentration of a given combination of arbitrary drug concentrations. The FIC values used here were calculated using the assumption of the Bliss Independence Model (Bliss, 1939), as this model enables us to calculate FIC values at different inhibition levels of the individual drugs. The Bliss Independence Model assumes that drugs act on cells through independent mechanisms and the combined drug effects are additive (Bliss, 1939). This means, the expected killing rate, $E_{A+B}$ , of two drugs A and B would be:

$$E_{A+B}=E_{A}+\left(1-E_{A}\right)\cdot E_{B}$$

where

$$E_{i}={\text{Emax}}_{\left\{\text{frac}\_\text{killed},i\right\}}\frac{{{\text{Conc}}_{\left\{N,i\right\}}}^{h_{i}}}{{{\text{Conc}}_{\left\{N,i\right\}}}^{h_{i}}+{{C50}_{\left\{N,i\right\}}}^{h_{i}}}$$ (15)

where i=A or B

We then calculate $E_{A+B}$ and derive ${\text{Conc}}_{eff}$ from the equation below:

$$E_{A+B}={\text{Emax}}_{\left\{\text{frac}\_\text{killed},\text{comb}\right\}}\frac{{{\text{Conc}}_{\text{eff}}}^{h_{\left\{comb,\exp\right\}}}}{{{\text{Conc}}_{\text{eff}}}^{h_{\left\{comb,\exp\right\}}}+{{C50}_{\left\{N,comb,\exp\right\}}}^{h_{\left\{comb,\exp\right\}}}}$$ (16)

where

$$h_{\left\{comb,\exp\right\}}=\frac{\log\left(\frac{2{\text{Emax}}_{\left\{\text{frac}\_\text{killed},\text{comb}\right\}}-1}{1.1{\text{Emax}}_{\left\{\text{frac}\_\text{killed},\text{comb}\right\}}-1}\right)}{\log\left(\frac{{BC90}_{\exp}}{{BC50}_{\exp}}\right)}$$ (17)

And

$${C50}_{\left\{N,comb,\exp\right\}}={\text{BC}50}_{\exp}{\left(2{\text{Emax}}_{\left\{\text{frac}\_\text{killed},\text{comb}\right\}}-1\right)}^{1/h_{\left\{comb,\exp\right\}}}$$ (18)

The equations for $h_{\left\{comb,\exp\right\}}$ and ${C50}_{\left\{N,comb,\exp\right\}}$ (Equations 15–18) are derived from the assumptions that the concentrations ${\text{BC}90}_{\exp}$ and ${\text{BC}50}_{\exp}$ lead to 90% and 50% bacterial killing, respectively, which can be defined from the Hill equation in Equations 19 and 20 as:

$$0.9={\text{Emax}}_{\left\{\text{frac}\_\text{killed},\text{comb}\right\}}\frac{{{\text{BC}90}_{\exp}}^{h_{\left\{comb,\exp\right\}}}}{{{\text{BC}90}_{\exp}}^{h_{\left\{comb,\exp\right\}}}+{{C50}_{\left\{N,comb,\exp\right\}}}^{h_{\left\{comb,\exp\right\}}}}$$ (19)

and

$$0.5={\text{Emax}}_{\left\{\text{frac}\_\text{killed},\text{comb}\right\}}\frac{{{\text{BC}50}_{\exp}}^{h_{\left\{comb,\exp\right\}}}}{{{\text{BC}50}_{\exp}}^{h_{\left\{comb,\exp\right\}}}+{{C50}_{\left\{N,comb,\exp\right\}}}^{h_{\left\{comb,\exp\right\}}}}$$ (20)

#### Calculating the killing rate of the combination of interest ( $k\left(C_{A}+C_{B}\right)$ )

Once we derive $h_{{\text{Comb}}_{\text{observed}}}$ , ${C50}_{\left\{N,comb,obs\right\}}$ , $E_{\max_{{\text{Comb}}_{\text{observed}}}}$ and $\text{Con}c_{\text{eff}}$ , we can calculate the killing rate of the combination drug A and drug B with the concentrations C_A_ and C_B_ as:

$$k\left(C_{A}+C_{B}\right)=E_{\max_{\left\{\text{comb},\text{obs}\right\}}}\;\frac{{{\text{Conc}}_{\text{eff}}}^{h_{\left\{comb,obs\right\}}}}{{{\text{Conc}}_{\text{eff}}}^{h_{\left\{comb,obs\right\}}}+{{C50}_{\left\{N,comb,obs\right\}}}^{h_{\left\{comb,obs\right\}}}}$$ (21)

Using Equation 21, we estimate the missing data from a checkerboard assay of drug-drug interaction (Figure 4, see Supplementary File 5 for killing rates of all drug combinations and concentrations).

##### Including *in vitro* measurements into *GranSim* to track PD and drug-drug interactions

For each drug combination, we generate lookup tables that contain the overall regimen killing rate corresponding to the collection of concentrations of each individual drug within the combination. We calculate the killing rates of each combination using the method described in Section 5 above. We use these lookup tables in *GranSim* to determine the killing rates ( k ) that each bacterium is exposed to within a simulation based on the spatial concentrations of drugs. Bacteria are killed on each timestep with probability $1-e^{-k}$ using a stochastic process.

### Antibiotic treatment simulations

Using *GranSim*, we simulated each regimen using our *in silico* granuloma library containing 100 high-CFU and 100 low-CFU granulomas, totaling 200 granulomas, for a maximum of 180 days (shorter simulations to be consistent when comparing to experimental/clinical studies are clarified below). Unless otherwise stated, we administer human or human-equivalent doses of each drug within a regimen daily, based on the model we used for PK calibration (Budak et al., 2023; Budak et al., 2024): 5 mg/kg of isoniazid (World Health Organization, 2014), 10 mg/kg of rifampicin (World Health Organization, 2014), 7 mg/kg of moxifloxacin (World Health Organization, 2014), 20 mg/kg of ethambutol (World Health Organization, 2014), 25 mg/kg of pyrazinamide (World Health Organization, 2014), 20 mg/kg of Bedaquiline (human-equivalent dose for rabbits), 20 mg/kg of pretomanid (human-equivalent dose for rabbits), and 90 mg/kg of linezolid (human-equivalent dose for rabbits) (Budak et al., 2024).

We performed three complete sets of antibiotic treatment simulations:

a. Treatment simulations to replicate clinically relevant regimens and to choose the right stress condition that models the PD of nonreplicating Mtb:We simulated regimens that have been widely studied in clinical and preclinical trials, namely, BPaMZ (Cevik et al., 2024; Tweed et al., 2019; Li et al., 2017; Xu et al., 2019), BPaL (Tasneen et al., 2016; Xu et al., 2019; Bigelow et al., 2020; Berry et al., 2022; Conradie et al., 2020; Conradie et al., 2022), HRZE (Burman et al., 2006; Gillespie et al., 2014; Dorman et al., 2009; Johnson et al., 2009), RMZE (Nuermberger et al., 2004a; Gillespie et al., 2014; Nuermberger et al., 2004b; Li et al., 2015; Dorman et al., 2009) and HRZM (Nuermberger et al., 2004a; Burman et al., 2006; Conde et al., 2009; Gillespie et al., 2014; Li et al., 2015), for 180 days using 15 different sets of *in vitro* measurements to model the PD and drug interaction against nonreplicating Mtb (Table 1).
b. Treatment simulations to replicate regimens used in marmoset studies (marmoset regimens):Marmosets were treated with RMZE, BPa, BPaL, BL, RM, HRZE, PaL, B, Pa, RZ, HZ, M, Z, R, H for 60 days (Budak et al., 2024). For validation, we simulated the same regimens for 60 days using the maximum of AN and NH as the PD and drug interaction model for nonreplicating Mtb chosen in the previous step (8a).
c. Treatment simulations with the regimens used within Phase IIb meta-analysis human studies (Bonnett et al., 2017) (clinical regimens):We simulated regimens for 8 weeks using the maximum of AN and NH as the PD and drug interaction model for nonreplicating Mtb. As the doses and dosing interval of these regimens are different than standard, we abbreviate the regimens with the one-letter abbreviation of the drugs within the regimen followed by the dose of that drug in mg/kg, with the doses per week (dpw) listed at the end of the regimen abbreviation, as we have done previously (Cicchese J. et al., 2020). For example, E45R23.5dpw2 means that we simulated dosing granulomas with 45 mg/kg of E and 23.5 mg/kg of R 2 days per week.

### Methods to evaluate regimen efficacy

#### Metabolic activity using 2-deoxy-2-[18F]-fluoro-D-glucose (FDG) avidity

Positron Emission Tomography and Computed Tomography (PET-CT) scans are widely used methods to assess the metabolic activity of granulomas in clinical or NHP studies by measuring the uptake of the glucose analog FDG (White et al., 2017). To compare to experimental studies, we quantify the metabolic activity in *GranSim* as a proxy for PET-CT scan measurements. This measure is called FDG avidity and considers pro- and anti-inflammatory activity within granulomas. We calculate FDG avidity of a granuloma at time *t* in *GranSim* as:

$${FDG\;avidity}_{t}=\sum_{k=1}^{n}{4M}_{i_{k}}+M_{ci_{k}}+{6M}_{a_{k}}+{3T}_{{gam}_{k}}+{3T}_{{cyt}_{k}}+{3T}_{{reg}_{k}}$$ (22)

where n is the number of microgrids on *GranSim*’s simulation grid, $M_{i}$ , $M_{ci}$ , $M_{a}$ , $T_{gam}$ , $T_{cyt}$ and $T_{reg}$ are the number of resting macrophages, infected macrophages, chronically infected macrophages, active macrophages, IFN-γ producing T cells, cytotoxic T cells and regulatory T cells at microgrid k, respectively. We modified this measure from (Budak et al., 2023) to account for anti-inflammatory activity.

We then measured the fold change in FDG avidity of a granuloma as:

$$Fold\;change\;in\;FDG\;avidity=\log_{2}\left(\frac{{FDG\;avidity}_{t2}+0.01}{{FDG\;avidity}_{t1}+0.01}\right)$$ (23)

Where t1 and t2 are a day before treatment starts and treatment end day, respectively.

#### Sterilization time

The effectiveness of a regimen depends on how quickly it can eliminate all CFUs within a granuloma. Therefore, we assessed the efficacy of a regimen based on the time needed for that regimen to kill all Mtb within a granuloma, i.e., sterilization time. Lower sterilization times of a regimen means higher efficacy and *vice versa*. If a regimen has not sterilized a granuloma by the end of the simulation, we assume the sterilization time of that regimen is the length of simulation. Our simulations are typically 6 months long that reflects standard treatment time. If we simulate regimens to compare to experimental datasets, then we adjust the treatment time to match experiments (see “Antibiotic treatment simulations” in Methods for details).

### Ranking regimens in a regimen set

A regimen set is a group of regimens to be ranked based on their simulated efficacies using *GranSim*. We assess regimen efficacies based on sterilization times of each granuloma and rank them accordingly. To rank the regimens in a regimen set, we calculate a ranking score for each regimen as described in Budak et al. (2024). We refer to the reference regimen as the regimen for which the ranking score is calculated. Briefly, we compare the sterilization times of the reference regimen pairwise to that of the rest of the regimens in the regimen set. We do this using a one-tailed t-test, assuming the measures are normally distributed. A regimen is considered more potent than a reference regimen if its sterilization times are significantly lower than that of the reference regimen, and *vice versa* (p < 0.05, one-tailed t-test). We then count the number of significantly more (and less) potent regimens than the reference regimen to calculate ranking score as:

$${\text{Ranking}\;\text{score}}_{\text{reference}\;\text{regimen}}=\left(\text{no}.\text{ of more potent }\text{regimens than the }\text{reference regimen}\right)-\left(\text{no}.\text{ of less }\text{potent regimens than }\text{the reference regimen}\right)$$ (24)

We calculate ranking scores of each regimen in a regimen set and used these scores to rank regimens, in that higher ranking scores and lower ranks mean the regimen performs better overall within that specific regimen set.

### Including the effect of bacterial burden into GEODE

To assess the impact of bacterial burden on drug killing rates (i.e., inoculum effect in *in vitro* studies), *M. tuberculosis* was cultured under a single-stressor assay, the butyrate condition. Cultures were grown to mid-log phase, then diluted to three starting bacterial load levels: OD_600_ of 0.05, 0.1, and 0.15 for the drug susceptibility assay (see Supplementary File 3 for the raw data). From these experiments, we derive Hill curve parameters ( $E_{\max}$ , C50 and h ) that yield drug kill rates dependent on drug concentrations for varying drugs (d) and bacterial load (OD600) (Figure 2). We define the drug effect as *kill ratio*, which indicates the ratio of the killed bacteria to total bacteria (i.e., the portion of the killed bacteria) depending on the drug concentration:

$$\text{Kill ratio}\left(C\right)=E_{\max}\frac{C^{h}}{C^{h}+{C50}^{h}}$$ (25)

Since the PK of each drug varies and the drug effect depends on drug concentration, we define *PK-relevant drug effect* $E_{\text{PK}}$ for each bacterial load and drug as:

$$E_{\text{PK}}\left(\text{OD}600,d\right)=\frac{{\text{Emax}}_{\text{OD}600,d}\int_{0}^{{\text{Cmax}}_{d}}\frac{C^{h_{\text{OD}600,d}}}{C^{h_{\text{OD}600,d}}+{{C50}_{\text{OD}600,d}}^{h_{\text{OD}600,d}}}\text{dC}}{{\text{Cmax}}_{d}}$$ (26)

where $E_{\text{ma}x_{\text{OD}600,d}}$ , $C50_{\text{OD}600,d}$ and $h_{\text{OD}600,d}$ are Hill curve parameters for drug d and bacterial load value of OD600, $C_{\text{ma}x_{d}}$ is the maximum concentration of drug d based on *GranSim* PK simulations (Supplementary Table S11; Supplementary Figure S4, see Figure 2 for the Hill curves of all drugs and bacterial loads).

We then convert OD600 values from bacterial culture to CFU concentrations (to make it consistent with *GranSim* which is based on CFU):

$$\text{CFU}/\text{ml}\left(\text{OD}600\right)=1.198\cdot 10^{8}/\text{ml}\cdot \text{OD}600+1.297\cdot 10^{6}/\text{ml}$$ (27)

OD-based CFU quantification (Equation 27) was based on standard microbiology technique where replicate Erdman-strain Mtb were grown at 37 °C/5% CO_2_ in liquid culture without agitation. The OD600 was measured with a spectrophotometer at different timepoints, and then dilutions were plated on 7H11 plates so that the CFU/mL could be calculated and compared against the OD from that culture.

**FIGURE 12:**
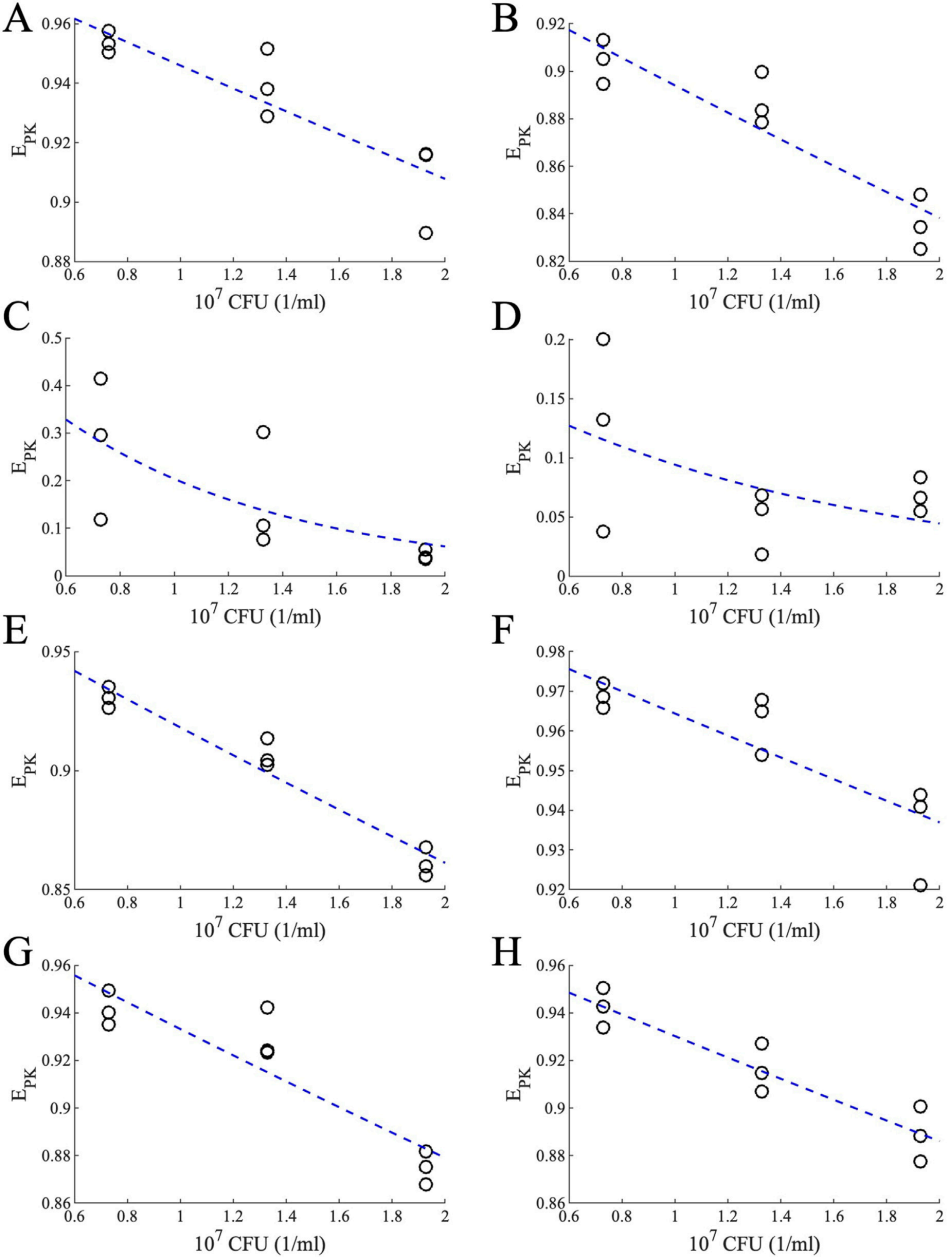
Effects of PK for different bacterial loads for different drugs. PK-relevant drug effect for different bacterial loads calculated using Equation 28 (black circles) and the exponential fit (blue dashed lines) for **(A)** INH, **(B)** RIF, **(C)** PZA, **(D)** EMB, **(E)** MXF, **(F)** BDQ, **(G)** PTM and **(H)** LZD.

Once we calculate CFU/mL values and their corresponding $E_{\text{PK}}$ values for each drug using Equation 26, we then fit these datapoints using an exponential function in the form of $Ae^{-Bx}$ such that:

$$E_{\text{PK}}\left(\frac{\text{CFU}}{\text{ml}},d\right)=A_{d}e^{{-B}_{d}\cdot \frac{\text{CFU}}{\text{ml}}}$$ (28)

where $A_{d}$ and $B_{d}$ are drug-dependent parameters (see Table 2; Figure 12).

**TABLE 2:** A and B values for all drugs (see Equation 25).

Drugs	A (unitless)	B (mL)
INH	0.99	4.1e−9
RIF	0.95	6.4e−9
PZA	0.67	1.2e−7
EMB	0.2	7.5e−8
MXF	0.98	6.4e−9
BDQ	0.99	2.9e−9
PTM	0.99	6e−9
LZD	0.98	4.9e−9

In *GranSim*, we calculate a modified killing rate $k_{\text{bacterial}\;load}$ by scaling the killing rate k (calculated in 5) using $E_{PK}$ values depending on CFU concentration:

$$k_{\text{bacterial}\;\text{load}}=k\cdot \frac{E_{\text{PK}}\left({\frac{\text{CFU}}{\text{ml}}}_{GranSim}\right)}{E_{\text{PK}}\left(\frac{\text{CFU}}{\text{ml}}\left(\text{OD}600=0.05\right)\right)}$$ (29)

where ${\frac{\text{CFU}}{\text{ml}}}_{GranSim}$ is the CFU concentration of simulated granulomas. In Equation 29, we normalize it to $E_{\text{PK}}\left(\frac{\text{CFU}}{\text{ml}}\left(\text{OD}600=0.05\right)\right)$ , as *in vitro* experiments (that were used to calculate k ) were performed using Mtb with OD600=0.05 .

When multiple drugs are available in a microgrid, g , in *GranSim*, then we calculate the bacterial load-dependent killing rate k at grid point g by taking the mean of $k_{\text{bacterial}\;load}$ values of all available drugs:

$$k_{{\text{bacterial}\;\text{load}}_{g}}=\frac{k\cdot \sum_{d=1}^{n}A_{d}e^{-B_{d}\cdot {\frac{\text{CFU}}{\text{ml}}}_{\text{GranSim}}}}{n{\cdot E}_{\text{PK}}\left(\frac{\text{CFU}}{\text{ml}}\left(\text{OD}600=0.05\right)\right)}$$ (30)

where n is the number of drugs available in the microgrid g .

### Drug treatment studies in macaques

Adult male Mauritian cynomolgus macaques were used in this study and obtained from Bioculture US. All experimental procedures involving care of animals complied with ethical regulations of the University of Pittsburgh School of Medicine Institutional Animal Care and Use Committee. Macaques were housed and cared for in accordance with local, state, federal, and institute policies in facilities accredited by the American Association for Accreditation of Laboratory Animal Care, under standards established in the Animal Welfare Act and the Guide for the Care and Use of Laboratory Animals as mandated by the U.S. Public Health Service Policy. Macaques were monitored for physical health, food consumption, body weight, temperature, complete blood counts, and serum chemistries. Examination of animals was performed in quarantine upon arrival at the University of Pittsburgh to assess physical health and confirm no previous Mtb infection was detected via ELISpot assays. All Mtb infections were performed a Biosafety Level 3 (BSL3) facility. Bronchoscopic instillation with Mtb Erdman was performed as previously described with an average dose of ∼19 CFU (Capuano et al., 2003; Lin et al., 2009). Veterinary staff regularly monitored clinical signs following challenge, including appetite, behavior and activity, weight, erythrocyte sedimentation rate, Mtb growth from gastric aspirate, and coughing.

Five adult male Mauritian cynomolgus macaques were obtained from Bioculture, Inc (Mauritius), with an age range of 5–9 years (Supplementary Material 1 provides all macaque data from the BPaL study). Macaques were co-housed in a Biosafety Level 3 facility and infected with 8–22 CFU *M. tuberculosis* strain Erdman via bronchoscope as described previously (Winchell et al., 2023). Approximately 8 weeks after *M. tuberculosis* infection, the animals were treated with the BPaL regimen daily via oral administration in food treats for 4 weeks. Drug doses: Bedaquiline (B) 40 mg/kg, Pretomanid (P) 62 mg/kg, Linezolid (L) 20 mg/kg. RMZE and HRZE treated animals were obtained from Valley Biosystems (5 adult males with age range of 4–9 years) and were infected with 8–21 CFU (Budak et al., 2023). Approximately 12 weeks after Mtb infection, the animals were treated with HRZE (n = 3) or RMZE (n = 2) for 8 weeks. Drug doses: Isoniazid (H) 15 mg/kg; Rifampicin (R) 20 mg/kg; Pyrazinamide (Z) 150 mg/kg; Ethambutol (E) 55 mg/kg; Moxifloxacin (M) 35 mg/kg. For all drug regimens, PET CT scans were performed immediately prior to initiation of drug treatment, and 4 and 8 weeks after initiation of drug treatment using 18-F fluorodeoxyglucose as a PET probe as previously described (White et al., 2017; Winchell et al., 2020). The BPaL-treated animals had only the 4 weeks post-drug treatment scan directly prior to necropsy with the shorter treatment time. The HRZE- and RMZE-treated animals had both a 4 weeks and 8 weeks scans. Granuloma size (measured by maximum diameter in mm) and avidity (measured by maximum SUV and corrected for granuloma size) were evaluated using OsiriX DICOM imaging software in the axial view. Avidity was measured for granulomas resulting in SUVR values pre- and post-drug treatment (White et al., 2017). A fold change was calculated per granuloma to measure drug effect on an individual granuloma basis (see Equation 31 below). At necropsy the final PET CT scan was used as a map to identify and individually isolate all granulomas or other pathologies, uninvolved lung lobes, thoracic lymph nodes and any signs of extrapulmonary disease. Each sample was plated for bacterial burden individually on 7H10 plates; the plates were incubated at 37° with 5% CO2 and counted for colony forming units (CFU) after 3 weeks. The fraction (%) of sterile (CFU negative) granulomas was calculated for each animal.

$$\text{Fold change in SUVR}=\log_{2}\left(\frac{\text{prenecropsy}\;\text{SUVR}+0.01\;}{\text{predrug}\;\text{SUVR}+0.01}\right)$$ (31)

## Data Availability

The raw data supporting the conclusions of this article will be made available by the authors, without undue reservation.
