# Supplementary material for: GEODE: an in silico tool that translates in vitro to in vivo predictions of tuberculosis antibiotic combination efficacy
Source: Front Pharmacol. 2025 Oct 17;16:1639673. doi: 10.3389/fphar.2025.1639673 (PMC12575316; doi:10.3389/fphar.2025.1639673)
Supplement: Supplementary file 4 [file Supplementaryfile1.zip › Glossary.DOCX]

Glossary

**Inhibitory concentration (IC_X_):** Drug concentrations needed to reach X level of growth inhibition level. The growth inhibition level X of a drug concentration C can be calculated using OD_600_ values of untreated ($\mathrm{OD}_{{600}_{\mathrm{untreated}}}$) and treated bacteria ($\mathrm{OD}_{{600}_{\mathrm{treated}}}$):

$\frac{X}{100}=\frac{\mathrm{OD}_{{600}_{\mathrm{treated}}}}{\mathrm{OD}_{{600}_{\mathrm{untreated}}}}$ (Eq. 1)

For example, if $X=50$, then the drug concentration $C$ is the $\mathrm{IC}_{50}$ of that drug, meaning that $C$ inhibits 50% growth in bacteria. $\mathrm{IC}_{X}$ can be derived using the Hill equation with growth inhibition-based Hill curve parameters:

$E\left( \mathrm{IC}_{X} \right)=E_{\max}\frac{{\mathrm{IC}_{X}}^{h}}{{\mathrm{IC}_{X}}^{h}+{C_{50}}^{h}}=X\%$ (Eq. 2)

Where $E$ (or $X\%$) is the level of growth inhibition, $E_{max}$ is the maximum growth inhibition level a drug can reach, $C_{50}$ is the concentration needed to reach the half maximum effect ($E_{max}$/2) and $h$ is the Hill constant.

**Bactericidal concentration (BC_X_):** Drug concentrations needed to reach killing level of X. For example, $\mathrm{BC}_{50}$ is the drug concentration that kills $50\%$ of bacteria. $\mathrm{BC}_{X}$ can be derived using the Hill equation with growth inhibition-based Hill curve parameters:

$E\left( \mathrm{BC}_{X} \right)=E_{\max}\frac{{\mathrm{BC}_{X}}^{h}}{{\mathrm{BC}_{X}}^{h}+{C50}^{h}}=X\%$ (Eq. 3)

Where $E$ (or $X\%$) is the level of bacterial killing, $E_{max}$ is the maximum bacterial killing level a drug can reach, $C_{50}$ is the concentration needed to reach the half maximum effect ($E_{max}$/2) and $h$ is the Hill constant.

**Drug interaction models:** Evaluating drug-drug interactions requires a universal reference model that describes the expected effect of multiple drug combinations to decide whether observed effects are synergistic or antagonistic. There are many models that have been used to assess drug interactions, with Bliss independence and Loewe additivity being the most accepted ones [1, 2], and in this study we assumed Bliss independence model describes drug interaction. According to the Bliss independence model, the effects of multiple drugs are additive, as it assumes that the combined drugs act on the cell/pathogen targets through independent mechanisms [1]. In contrast, the Loewe additivity model assumes that drugs cannot interact with each other and also that drugs act on the cells/pathogen targets through the same mechanism [2]. Thus, Loewe additivity assumes the doses of individual drugs are additive. The drug interaction field was lacking agreement between the existing models, until recently when a consensus framework, Multi-dimensional Synergy of Combinations (MuSyc), was introduced. MuSyc is a unifying framework that uses the Law of Mass Action to unify different models of drug interactions [3].

**Fractional inhibitory concentration (FIC{X}):** A standard metric for scoring drug interactions using growth inhibition measures. It is used to quantify curvature of contours for common phenotypes in a checkerboard assay and is calculated as

$FIC\{X\}=\frac{{IC\{X\}}_{\mathrm{obs}}}{{IC\{X\}}_{\exp}}$, (Eq. 4)

where $X$ is the phenotype of interest (e.g., $50\%$ growth inhibition), ${IC\{X\}}_{\mathrm{obs}}$ and ${IC\{X\}}_{\exp}$ are the observed and expected inhibitory concentrations to reach $X$. ${\mathrm{IC}\{X\}}_{\exp}$ is determined based on the reference drug interaction model (e.g., Loewe additivity, Bliss independence). Non-interacting drugs have an FIC of 1, whereas synergistic and antagonistic drugs have FICs lower- and higher than 1, respectively.

**Fractional bactericidal concentration (FBC_X_):** A standard metric for scoring drug interactions using bactericidal measures. It is used to quantify curvature of contours for common phenotypes in a checkerboard and is calculated as

$FBC\{X\}=\frac{{BC\{X\}}_{\mathrm{obs}}}{{BC\{X\}}_{\exp}}$ (Eq. 5)

where $X$ is the phenotype of interest (e.g., $50\%$ bacterial killing), ${\mathrm{BC}\{X\}}_{\mathrm{obs}}$ and ${\mathrm{BC}\{X\}}_{\exp}$ are the observed and expected (respective) bactericidal concentrations to reach $X$. ${\mathrm{BC}\{X\}}_{\exp}$ is determined based on a reference drug interaction model (e.g., Loewe additivity, Bliss independence, see above). Non-interacting drugs have an FBC of 1, whereas synergistic and antagonistic drugs have FBCs lower- and higher than 1, respectively.

**Fractional survival (**$\mathrm{CFU}_{\{frac\_survival, X\}}$**):** Fraction of live bacteria remaining after treatment with $IC_{X}$. For example, if a drug concentration inhibits $X\%$ of bacterial growth and kills $Y\%$ of bacteria, then $\mathrm{CFU}_{\{frac\_survival, X\}}$ is $(100-Y)\%$ and $IC_{X}=BC_{Y}$.

**Growth inhibition-based parameters for single drugs and combinations**

${E\max}_{\mathrm{inh}}$: Maximum growth inhibition level a drug can reach

$C{50}_{\mathrm{inh}}$ : $C50$ is the concentration needed to reach the half maximum growth inhibition level ($\mathrm{Emax}$/2)

$h_{\mathrm{inh}}$: growth inhibition-based Hill constant

**Bactericidal activity-based parameters for single drugs and combinations**

*Single drug parameters*

${\mathrm{BC}\{X\}}_{A}$: $\mathrm{BC}$ of drug A to reach killing level of $X$

${E\max}_{A}$: Bactericidal activity-based $\mathrm{Emax}$ for drug A

${C50}_{\{N,A\}}$: Bactericidal activity-based $C50$ for drug A normalized to ${\mathrm{BC}90}_{A}$

$h_{A}$: Bactericidal activity-based $h$ for drug A

*Combination parameters*

$\mathrm{Emax}_{\{frac\_killed,A\}}$: Maximal fraction of killed bacteria for drug A

${E\max}_{\mathrm{comb}}$: Bactericidal activity-based $\mathrm{Emax}$ for the equipotent combination

${E\max}_{\{frac\_killed,comb\}}$: Maximal fraction of killed bacteria using equipotent combination

${\mathrm{FBC}\{X\}}_{Diag}$: FBC value of $BC_{X}$ for the equipotent combination (diagonal of the checkerboard)

**Parameters used to calculate drug effect for off-diagonal combinations**

*Single drug parameters*

$C_{A}$ : concentration of drug A

$\mathrm{Conc}_{\{N,A\}}$: Concentration of drug A normalized to ${\mathrm{BC}90}_{A}$($C_{A}$ /${BC90}_{A}$)

${BC\{X\}}_{\{N,A\}}$: BC of drug A to reach killing level of $X$ normalized to $\mathrm{BC}_{{90}_{A}}$

*Combination parameters*

${E\max}_{\{comb,obs\}}$: Bactericidal activity-based Emax for the combination of interest

${\mathrm{BC}\{X\}}_{\mathrm{obs}}$: BC of the combination of interest to reach killing level of X

${\mathrm{BC}\{X\}}_{\exp}$: BC of the combination of interest to reach killing level of X if there were no drug interaction involved

${\mathrm{FBC}\{X\}}_{\mathrm{obs}}$: $\mathrm{FBC}$ value of $BC_{X}$ for the combination of interest

$\mathrm{Conc}_{\mathrm{diff}}$: Concentration difference between two drugs normalized to the sum of concentrations. This parameter varies between 0 (at the diagonal) and 1 (single drug cases at x- and y-axis).

$h_{\{comb,obs\}}$: Bactericidal activity-based $h$ for the combination of interest

$h_{\{comb,exp\}}$: Bactericidal activity-based $h$ for the combination of interest if there are no drug-drug interactions involved

${C50}_{\{N,comb,\mathrm{obs}\}}$: Bactericidal activity-based $C50$ for the combination of interest normalized to ${BC90}_{\mathrm{obs}}$

${C50}_{\{N,comb,exp\}}$: Bactericidal activity-based $C50$ for the combination of interest normalized to ${\mathrm{BC}90}_{\mathrm{obs}}$ if there are no drug-drug interactions involved

$\mathrm{Con}c_{\mathrm{eff}}$: Effective concentration of a combination

$k\left( C_{A} \right)$: killing rate of drug A with concentration $C$

**References**

1. C.I.Bliss. THE TOXICITY OF POISONS APPLIED JOINTLY. Annals of Applied Biology. 1939;26(3):585-615.

2. Muischnek SLaH. Über Kombinationswirkungen. Archiv f experiment Pathol u Pharmakol. 1926;114:313-26. doi: <https://doi.org/10.1007/BF01952257>.

3. Wooten DJ, Meyer CT, Lubbock ALR, Quaranta V, Lopez CF. MuSyC is a consensus framework that unifies multi-drug synergy metrics for combinatorial drug discovery. Nature communications. 2021;12(1):4607. Epub 20210729. doi: 10.1038/s41467-021-24789-z. PubMed PMID: 34326325; PubMed Central PMCID: PMCPMC8322415.
