# Supplementary material for: GEODE: an in silico tool that translates in vitro to in vivo predictions of tuberculosis antibiotic combination efficacy
Source: Front Pharmacol. 2025 Oct 17;16:1639673. doi: 10.3389/fphar.2025.1639673 (PMC12575316; doi:10.3389/fphar.2025.1639673)
Supplement: Supplementary file 4 [file Supplementaryfile1.zip › Supplementary Figures and Tables.docx]

**
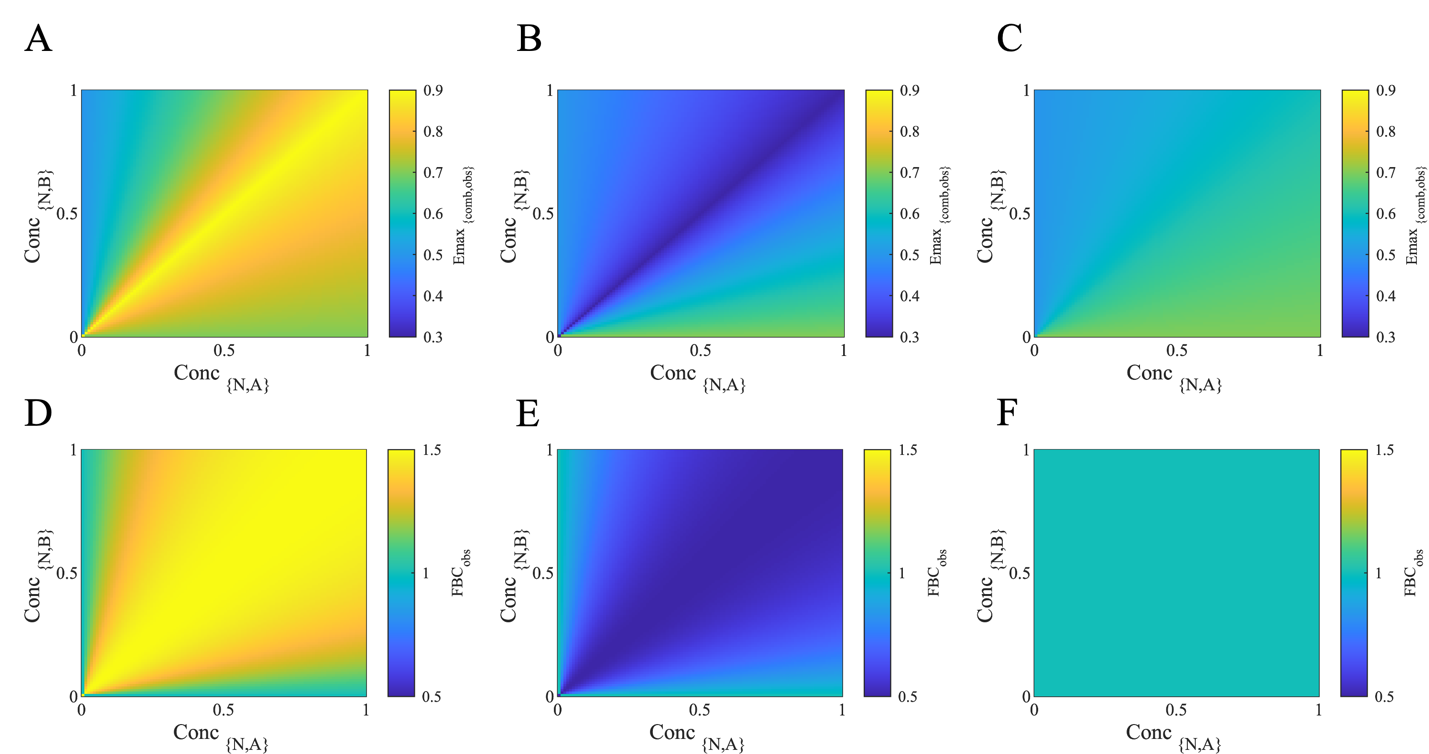
Supplementary Figure 1:** **Drug concentrations for different conditions**. (A-C) ${E\max}_{\{comb,obs\}}$ and (D-F) $\mathrm{FB}C_{\mathrm{obs}}$ values of various normalized concentrations of 2 drugs for synergistic [A (${E\max}_{\mathrm{comb}}$= 0.9) and D ($\mathrm{FB}C_{D\mathrm{iag}}$ = 1.5)], antagonistic [B (${E\max}_{\mathrm{comb}}$= 0.3) and E ($\mathrm{FB}C_{\mathrm{diag}}$ = 0.5)] and additive [C (${E\max}_{\mathrm{comb}}$= 0.5) and F ($\mathrm{FB}C_{D\mathrm{iag}}$ = 1)] drug combinations with $\mathrm{Emax}_{A}=0.7$ and $\mathrm{Emax}_{B}=0.5$.


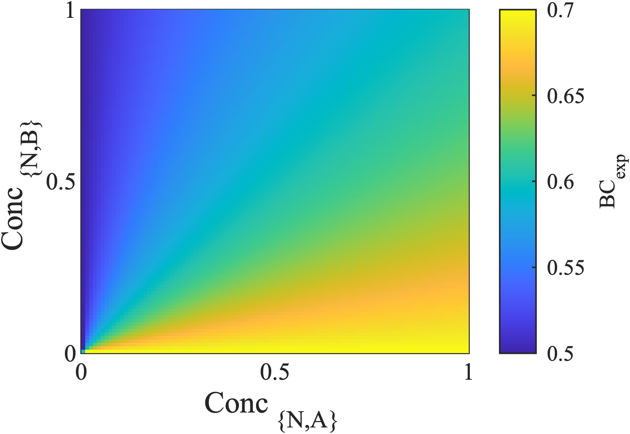


**Supplementary Figure 2: BC_exp_ values for various normalized concentrations of 2 drugs with** $\mathbf{BC}_{\boldsymbol{\{N,A\}}}$**= 0.7 and** $\mathbf{BC}_{\boldsymbol{\{N,B\}}}$**= 0.5.**


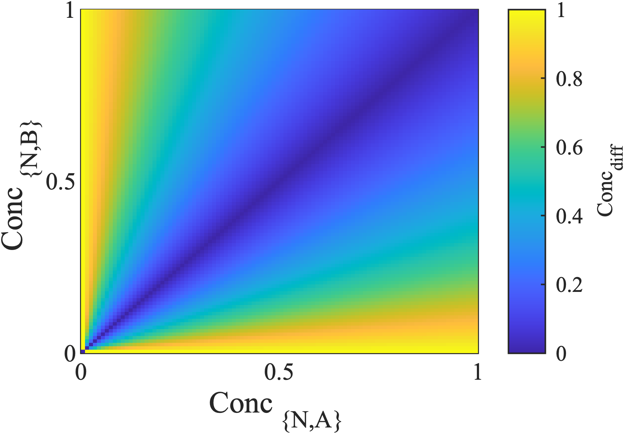


**Supplementary Figure 3:** $\mathbf{Con}\mathbf{c}_{\mathbf{diff}}$ **values for various normalized concentrations of 2 drugs.**


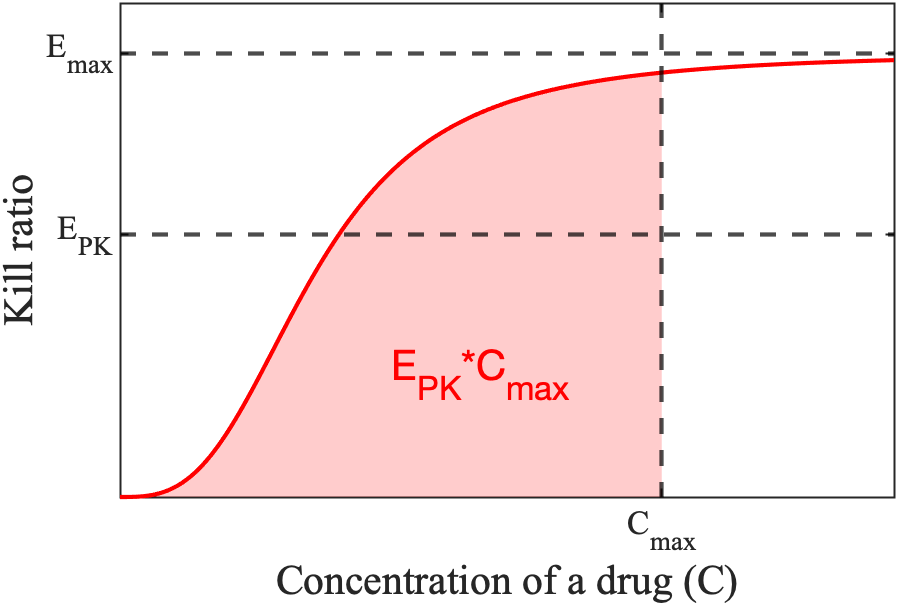


**Supplementary Figure 4: Curve of kill ratios by drug concentrations**. $E_{\mathrm{PK}}$ measurement using Hill curve parameters from bacterial load experiments and $C_{\max}$ values from *GranSim* simulations ($E_{\mathrm{PK}}$: PK-relevant drug effect, $E_{\max}$: maximum drug effect, $C_{\max}$: maximum concentration of a drug in *GranSim* simulations).

**Supplementary Table 1: *GranSim* rankings of marmoset regimens considering all granulomas**

| **Reference regimen** | **Rank** | **Nr of regimens significantly worse than the ref regimen (w)** | **Reference regimen is statistically better than** | **Nr of regimens significantly better than the ref regimen (b)** | **Reference regimen is statistically worse than** | **Ranking score (w-b)** | **Reference regimen is not significantly different than** |
| --- | --- | --- | --- | --- | --- | --- | --- |
| **BPaL** | 1 | 12 | HRZE,RMZE,BL,H,HZ,PaL,R,Z,B,M,RM,RZ | 0 |  | 12 | BPa,Pa |
| **BPa** | 1 | 12 | HRZE,RMZE,BL,H,HZ,PaL,R,Z,B,M,RM,RZ | 0 |  | 12 | BPaL,Pa |
| **Pa** | 1 | 12 | HRZE,RMZE,BL,H,HZ,PaL,R,Z,B,M,RM,RZ | 0 |  | 12 | BPaL,BPa |
| **RM** | 4 | 9 | RMZE,BL,H,HZ,PaL,R,Z,M,RZ | 3 | BPaL,BPa,Pa | 6 | HRZE,B |
| **HRZE** | 5 | 8 | RMZE,BL,H,HZ,PaL,Z,M,RZ | 3 | BPaL,BPa,Pa | 5 | R,B,RM |
| **B** | 5 | 8 | RMZE,BL,H,HZ,PaL,Z,M,RZ | 3 | BPaL,BPa,Pa | 5 | HRZE,R,RM |
| **R** | 7 | 6 | H,HZ,PaL,Z,M,RZ | 4 | BPaL,BPa,Pa,RM | 2 | HRZE,RMZE,BL,B |
| **RMZE** | 8 | 6 | H,HZ,PaL,Z,M,RZ | 6 | HRZE,BPaL,BPa,B,Pa,RM | 0 | BL,R |
| **BL** | 8 | 6 | H,HZ,PaL,Z,M,RZ | 6 | HRZE,BPaL,BPa,B,Pa,RM | 0 | RMZE,R |
| **HZ** | 10 | 4 | H,PaL,Z,M | 9 | HRZE,BPaL,RMZE,BPa,BL,R,B,Pa,RM | -5 | RZ |
| **RZ** | 10 | 4 | H,PaL,Z,M | 9 | HRZE,BPaL,RMZE,BPa,BL,R,B,Pa,RM | -5 | HZ |
| **PaL** | 12 | 3 | H,Z,M | 11 | HRZE,BPaL,RMZE,BPa,BL,HZ,R,B,Pa,RM,RZ | -8 |  |
| **H** | 13 | 1 | Z | 12 | HRZE,BPaL,RMZE,BPa,BL,HZ,PaL,R,B,Pa,RM,RZ | -11 | M |
| **M** | 13 | 1 | Z | 12 | HRZE,BPaL,RMZE,BPa,BL,HZ,PaL,R,B,Pa,RM,RZ | -11 | H |
| **Z** | 15 | 0 |  | 14 | HRZE,BPaL,RMZE,BPa,BL,H,HZ,PaL,R,B,M,Pa,RM,RZ | -14 |  |

**Supplementary Table 2: *GranSim* rankings of marmoset regimens considering low-CFU granulomas only**

| **Reference regimen** | **Rank** | **Nr of regimens significantly worse than the ref regimen (w)** | **Reference regimen is statistically better than** | **Nr of regimens significantly better than the ref regimen (b)** | **Reference regimen is statistically worse than** | **Ranking score (w-b)** | **Reference regimen is not significantly different than** |
| --- | --- | --- | --- | --- | --- | --- | --- |
| **BPa** | 1 | 13 | HRZE,BPaL,RMZE,BL,H,HZ,PaL,R,Z,B,M,RM,RZ | 0 |  | 13 | Pa |
| **Pa** | 2 | 11 | HRZE,RMZE,BL,H,HZ,PaL,R,Z,M,RM,RZ | 0 |  | 11 | BPaL,BPa,B |
| **BPaL** | 3 | 10 | HRZE,BL,H,HZ,PaL,R,Z,M,RM,RZ | 1 | BPa | 9 | RMZE,B,Pa |
| **B** | 4 | 8 | BL,H,HZ,PaL,R,Z,M,RZ | 1 | BPa | 7 | HRZE,BPaL,RMZE,Pa,RM |
| **RMZE** | 5 | 8 | BL,H,HZ,PaL,R,Z,M,RZ | 2 | BPa,Pa | 6 | HRZE,BPaL,B,RM |
| **RM** | 6 | 8 | BL,H,HZ,PaL,R,Z,M,RZ | 3 | BPaL,BPa,Pa | 5 | HRZE,RMZE,B |
| **HRZE** | 7 | 7 | H,HZ,PaL,R,Z,M,RZ | 3 | BPaL,BPa,Pa | 4 | RMZE,BL,B,RM |
| **BL** | 8 | 6 | H,HZ,PaL,Z,M,RZ | 6 | BPaL,RMZE,BPa,B,Pa,RM | 0 | HRZE,R |
| **R** | 9 | 6 | H,HZ,PaL,Z,M,RZ | 7 | HRZE,BPaL,RMZE,BPa,B,Pa,RM | -1 | BL |
| **HZ** | 10 | 4 | H,PaL,Z,M | 9 | HRZE,BPaL,RMZE,BPa,BL,R,B,Pa,RM | -5 | RZ |
| **RZ** | 10 | 4 | H,PaL,Z,M | 9 | HRZE,BPaL,RMZE,BPa,BL,R,B,Pa,RM | -5 | HZ |
| **PaL** | 12 | 3 | H,Z,M | 11 | HRZE,BPaL,RMZE,BPa,BL,HZ,R,B,Pa,RM,RZ | -8 |  |
| **H** | 13 | 1 | Z | 12 | HRZE,BPaL,RMZE,BPa,BL,HZ,PaL,R,B,Pa,RM,RZ | -11 | M |
| **M** | 13 | 1 | Z | 12 | HRZE,BPaL,RMZE,BPa,BL,HZ,PaL,R,B,Pa,RM,RZ | -11 | H |
| **Z** | 15 | 0 |  | 14 | HRZE,BPaL,RMZE,BPa,BL,H,HZ,PaL,R,B,M,Pa,RM,RZ | -14 |  |

**Supplementary Table 3: *GranSim* rankings of marmoset regimens considering high-CFU granulomas only**

| **Reference regimen** | **Rank** | **Nr of regimens significantly worse than the ref regimen (w)** | **Reference regimen is statistically better than** | **Nr of regimens significantly better than the ref regimen (b)** | **Reference regimen is statistically worse than** | **Ranking score (w-b)** | **Reference regimen is not significantly different than** |
| --- | --- | --- | --- | --- | --- | --- | --- |
| **BPa** | 1 | 13 | HRZE,RMZE,BL,H,HZ,PaL,R,Z,B,M,Pa,RM,RZ | 0 |  | 13 | BPaL |
| **BPaL** | 2 | 12 | HRZE,RMZE,BL,H,HZ,PaL,R,Z,B,M,RM,RZ | 0 |  | 12 | BPa,Pa |
| **Pa** | 3 | 12 | HRZE,RMZE,BL,H,HZ,PaL,R,Z,B,M,RM,RZ | 1 | BPa | 11 | BPaL |
| **RM** | 4 | 11 | HRZE,RMZE,BL,H,HZ,PaL,R,Z,B,M,RZ | 3 | BPaL,BPa,Pa | 8 |  |
| **HRZE** | 5 | 8 | RMZE,BL,H,HZ,PaL,Z,M,RZ | 4 | BPaL,BPa,Pa,RM | 4 | R,B |
| **R** | 5 | 8 | RMZE,BL,H,HZ,PaL,Z,M,RZ | 4 | BPaL,BPa,Pa,RM | 4 | HRZE,B |
| **B** | 5 | 8 | RMZE,BL,H,HZ,PaL,Z,M,RZ | 4 | BPaL,BPa,Pa,RM | 4 | HRZE,R |
| **BL** | 8 | 6 | H,HZ,PaL,Z,M,RZ | 7 | HRZE,BPaL,BPa,R,B,Pa,RM | -1 | RMZE |
| **RMZE** | 9 | 5 | H,PaL,Z,M,RZ | 7 | HRZE,BPaL,BPa,R,B,Pa,RM | -2 | BL,HZ |
| **HZ** | 10 | 4 | H,PaL,Z,M | 8 | HRZE,BPaL,BPa,BL,R,B,Pa,RM | -4 | RMZE,RZ |
| **RZ** | 11 | 4 | H,PaL,Z,M | 9 | HRZE,BPaL,RMZE,BPa,BL,R,B,Pa,RM | -5 | HZ |
| **H** | 12 | 2 | Z,M | 11 | HRZE,BPaL,RMZE,BPa,BL,HZ,R,B,Pa,RM,RZ | -9 | PaL |
| **PaL** | 12 | 2 | Z,M | 11 | HRZE,BPaL,RMZE,BPa,BL,HZ,R,B,Pa,RM,RZ | -9 | H |
| **Z** | 14 | 0 |  | 13 | HRZE,BPaL,RMZE,BPa,BL,H,HZ,PaL,R,B,Pa,RM,RZ | -13 | M |
| **M** | 14 | 0 |  | 13 | HRZE,BPaL,RMZE,BPa,BL,H,HZ,PaL,R,B,Pa,RM,RZ | -13 | Z |

**Supplementary Table 4: Rankings of marmoset experiments considering all granulomas from [1]**

| **Reference regimen** | **Rank** | **Nr of regimens significantly worse than the ref regimen (w)** | **Reference regimen is statistically better than** | **Nr of regimens significantly better than the ref regimen (b)** | **Reference regimen is statistically worse than** | **Ranking score (w-b)** | **Reference regimen is not significantly different than** |
| --- | --- | --- | --- | --- | --- | --- | --- |
| **BPaL** | 1 | 13 | RMZE,H,M,R,Z,HZ,RZ,RM,Pa,BPa,B,BL,PaL | 0 |  | 13 | HRZE |
| **HRZE** | 2 | 11 | H,M,R,Z,HZ,RZ,RM,Pa,B,BL,PaL | 0 |  | 11 | RMZE,BPa,BPaL |
| **BPa** | 3 | 11 | H,M,R,Z,HZ,RZ,RM,Pa,B,BL,PaL | 1 | BPaL | 10 | RMZE,HRZE |
| **RMZE** | 4 | 10 | H,M,R,Z,HZ,RZ,Pa,B,BL,PaL | 1 | BPaL | 9 | HRZE,RM,BPa |
| **RM** | 5 | 9 | H,M,R,Z,HZ,Pa,B,BL,PaL | 3 | HRZE,BPa,BPaL | 6 | RMZE,RZ |
| **RZ** | 6 | 9 | H,M,R,Z,HZ,Pa,B,BL,PaL | 4 | RMZE,HRZE,BPa,BPaL | 5 | RM |
| **M** | 7 | 4 | H,Z,Pa,PaL | 6 | RMZE,HRZE,RZ,RM,BPa,BPaL | -2 | R,HZ,B,BL |
| **R** | 7 | 4 | H,Z,Pa,PaL | 6 | RMZE,HRZE,RZ,RM,BPa,BPaL | -2 | M,HZ,B,BL |
| **HZ** | 7 | 4 | H,Z,Pa,PaL | 6 | RMZE,HRZE,RZ,RM,BPa,BPaL | -2 | M,R,B,BL |
| **B** | 7 | 4 | H,Z,Pa,PaL | 6 | RMZE,HRZE,RZ,RM,BPa,BPaL | -2 | M,R,HZ,BL |
| **BL** | 7 | 4 | H,Z,Pa,PaL | 6 | RMZE,HRZE,RZ,RM,BPa,BPaL | -2 | M,R,HZ,B |
| **Pa** | 12 | 2 | H,Z | 11 | RMZE,HRZE,M,R,HZ,RZ,RM,BPa,B,BPaL,BL | -9 | PaL |
| **PaL** | 12 | 2 | H,Z | 11 | RMZE,HRZE,M,R,HZ,RZ,RM,BPa,B,BPaL,BL | -9 | Pa |
| **H** | 14 | 0 |  | 13 | RMZE,HRZE,M,R,HZ,RZ,RM,Pa,BPa,B,BPaL,BL,PaL | -13 | Z |
| **Z** | 14 | 0 |  | 13 | RMZE,HRZE,M,R,HZ,RZ,RM,Pa,BPa,B,BPaL,BL,PaL | -13 | H |

**Supplementary Table 5: Rankings of marmoset experiments considering only fibrotic granulomas from [1]**

| **Reference regimen** | **Rank** | **Nr of regimens significantly worse than the ref regimen (w)** | **Reference regimen is statistically better than** | **Nr of regimens significantly better than the ref regimen (b)** | **Reference regimen is statistically worse than** | **Ranking score (w-b)** | **Reference regimen is not significantly different than** |
| --- | --- | --- | --- | --- | --- | --- | --- |
| **BPaL** | 1 | 12 | RMZE,H,M,R,Z,HZ,RZ,RM,Pa,B,BL,PaL | 0 |  | 12 | HRZE,BPa |
| **BPa** | 2 | 11 | H,M,R,Z,HZ,RZ,RM,Pa,B,BL,PaL | 0 |  | 11 | RMZE,HRZE,BPaL |
| **HRZE** | 3 | 9 | H,M,R,Z,HZ,RZ,RM,Pa,PaL | 0 |  | 9 | RMZE,BPa,B,BPaL,BL |
| **RMZE** | 4 | 6 | H,R,Z,HZ,Pa,PaL | 1 | BPaL | 5 | HRZE,M,RZ,RM,BPa,B,BL |
| **B** | 5 | 4 | H,Z,Pa,PaL | 0 |  | 4 | RMZE,HRZE,M,R,HZ,RZ,RM,BPa,BPaL,BL |
| **BL** | 5 | 4 | H,Z,Pa,PaL | 0 |  | 4 | RMZE,HRZE,M,R,HZ,RZ,RM,BPa,B,BPaL |
| **RM** | 7 | 5 | H,Z,HZ,Pa,PaL | 3 | HRZE,BPa,BPaL | 2 | RMZE,M,R,RZ,B,BL |
| **M** | 8 | 4 | H,Z,Pa,PaL | 3 | HRZE,BPa,BPaL | 1 | RMZE,R,HZ,RZ,RM,B,BL |
| **RZ** | 9 | 3 | H,Z,PaL | 3 | HRZE,BPa,BPaL | 0 | RMZE,M,R,HZ,RM,Pa,B,BL |
| **R** | 10 | 3 | H,Z,PaL | 4 | RMZE,HRZE,BPa,BPaL | -1 | M,HZ,RZ,RM,Pa,B,BL |
| **HZ** | 11 | 2 | H,PaL | 5 | RMZE,HRZE,RM,BPa,BPaL | -3 | M,R,Z,RZ,Pa,B,BL |
| **Pa** | 12 | 0 |  | 8 | RMZE,HRZE,M,RM,BPa,B,BPaL,BL | -8 | H,R,Z,HZ,RZ,PaL |
| **Z** | 13 | 0 |  | 10 | RMZE,HRZE,M,R,RZ,RM,BPa,B,BPaL,BL | -10 | H,HZ,Pa,PaL |
| **H** | 14 | 0 |  | 11 | RMZE,HRZE,M,R,HZ,RZ,RM,BPa,B,BPaL,BL | -11 | Z,Pa,PaL |
| **PaL** | 14 | 0 |  | 11 | RMZE,HRZE,M,R,HZ,RZ,RM,BPa,B,BPaL,BL | -11 | H,Z,Pa |

**Supplementary Table 6: Rankings of marmoset experiments considering only necrotic granulomas from [1]**

| **Reference regimen** | **Rank** | **Nr of regimens significantly worse than the ref regimen (w)** | **Reference regimen is statistically better than** | **Nr of regimens significantly better than the ref regimen (b)** | **Reference regimen is statistically worse than** | **Ranking score (w-b)** | **Reference regimen is not significantly different than** |
| --- | --- | --- | --- | --- | --- | --- | --- |
| **BPaL** | 1 | 13 | HRZE,H,M,R,Z,HZ,RZ,RM,Pa,BPa,B,BL,PaL | 0 |  | 13 | RMZE |
| **RMZE** | 2 | 11 | H,M,R,Z,HZ,RZ,RM,Pa,B,BL,PaL | 0 |  | 11 | HRZE,BPa,BPaL |
| **BPa** | 2 | 12 | HRZE,H,M,R,Z,HZ,RZ,RM,Pa,B,BL,PaL | 1 | BPaL | 11 | RMZE |
| **HRZE** | 4 | 9 | H,M,R,Z,HZ,Pa,B,BL,PaL | 2 | BPa,BPaL | 7 | RMZE,RZ,RM |
| **RZ** | 5 | 9 | H,M,R,Z,HZ,Pa,B,BL,PaL | 3 | RMZE,BPa,BPaL | 6 | HRZE,RM |
| **RM** | 6 | 7 | H,R,Z,Pa,B,BL,PaL | 3 | RMZE,BPa,BPaL | 4 | HRZE,M,HZ,RZ |
| **M** | 7 | 3 | H,Z,PaL | 5 | RMZE,HRZE,RZ,BPa,BPaL | -2 | R,HZ,RM,Pa,B,BL |
| **R** | 8 | 3 | H,Z,PaL | 6 | RMZE,HRZE,RZ,RM,BPa,BPaL | -3 | M,HZ,Pa,B,BL |
| **HZ** | 8 | 2 | H,PaL | 5 | RMZE,HRZE,RZ,BPa,BPaL | -3 | M,R,Z,RM,Pa,B,BL |
| **B** | 8 | 3 | H,Z,PaL | 6 | RMZE,HRZE,RZ,RM,BPa,BPaL | -3 | M,R,HZ,Pa,BL |
| **BL** | 8 | 3 | H,Z,PaL | 6 | RMZE,HRZE,RZ,RM,BPa,BPaL | -3 | M,R,HZ,Pa,B |
| **Pa** | 12 | 2 | H,PaL | 6 | RMZE,HRZE,RZ,RM,BPa,BPaL | -4 | M,R,Z,HZ,B,BL |
| **Z** | 13 | 0 |  | 10 | RMZE,HRZE,M,R,RZ,RM,BPa,B,BPaL,BL | -10 | H,HZ,Pa,PaL |
| **PaL** | 14 | 1 | H | 12 | RMZE,HRZE,M,R,HZ,RZ,RM,Pa,BPa,B,BPaL,BL | -11 | Z |
| **H** | 15 | 0 |  | 13 | RMZE,HRZE,M,R,HZ,RZ,RM,Pa,BPa,B,BPaL,BL,PaL | -13 | Z |

**Supplementary Table 7: *GranSim* rankings of clinical regimens considering all granulomas**

| **Reference regimen** | **Rank** |
| --- | --- |
| **H6R11dpw7** | 1 |
| **E16H6R10Z27dpw7** | 2 |
| **E25H6R10Z33dpw7** | 2 |
| **M7H5R10Z25dpw6** | 4 |
| **E17.5H5R10Z25dpw6** | 5 |
| **H11R10dpw5** | 5 |
| **R12.5dpw7** | 5 |
| **E15M7R10Z25dpw7** | 8 |
| **E29H14R13Z47dpw3** | 9 |
| **M9H14R10Z34dpw3** | 9 |
| **H6R10Z28dpw7** | 11 |
| **P2M7Z27dpw7** | 11 |
| **E16H6R9Z24dpw3** | 13 |
| **H16R12Z49dpw3** | 14 |
| **E16H6R10dpw7** | 15 |
| **E25H6R10dpw7** | 15 |
| **E25H10R10dpw7** | 15 |
| **E10H6R6dpw7** | 18 |
| **E25R23.5dpw7** | 19 |
| **E25R11dpw7** | 20 |
| **H6dpw7** | 20 |
| **E6H6dpw7** | 22 |
| **E15H6dpw7** | 22 |
| **E25H6dpw7** | 22 |
| **E25H10dpw7** | 22 |
| **E40H15R10dpw2** | 22 |
| **E90R24Z65.5dpw1** | 22 |
| **E45R23.5dpw2** | 28 |
| **E90R23.5dpw1** | 28 |

**Supplementary Table 8: *GranSim* rankings of clinical regimens considering low-CFU granulomas only**

| **Reference regimen** | **Rank** |
| --- | --- |
| **M7H5R10Z25dpw6** | 1 |
| **E15M7R10Z25dpw7** | 2 |
| **H6R11dpw7** | 2 |
| **E16H6R10Z27dpw7** | 4 |
| **E25H6R10Z33dpw7** | 4 |
| **E17.5H5R10Z25dpw6** | 6 |
| **R12.5dpw7** | 7 |
| **H11R10dpw5** | 8 |
| **M9H14R10Z34dpw3** | 9 |
| **P2M7Z27dpw7** | 10 |
| **E29H14R13Z47dpw3** | 11 |
| **H6R10Z28dpw7** | 12 |
| **E16H6R9Z24dpw3** | 13 |
| **H16R12Z49dpw3** | 14 |
| **E16H6R10dpw7** | 15 |
| **E25H6R10dpw7** | 15 |
| **E25H10R10dpw7** | 15 |
| **E10H6R6dpw7** | 18 |
| **E25R23.5dpw7** | 19 |
| **E25R11dpw7** | 20 |
| **H6dpw7** | 20 |
| **E6H6dpw7** | 22 |
| **E15H6dpw7** | 22 |
| **E25H6dpw7** | 22 |
| **E25H10dpw7** | 22 |
| **E40H15R10dpw2** | 22 |
| **E90R24Z65.5dpw1** | 22 |
| **E45R23.5dpw2** | 28 |
| **E90R23.5dpw1** | 28 |

**Supplementary Table 9: *GranSim* rankings of clinical regimens considering high-CFU granulomas only**

| **Reference regimen** | **Rank** |
| --- | --- |
| **H6R11dpw7** | 1 |
| **E16H6R10Z27dpw7** | 2 |
| **E25H6R10Z33dpw7** | 2 |
| **H11R10dpw5** | 4 |
| **R12.5dpw7** | 4 |
| **E17.5H5R10Z25dpw6** | 6 |
| **M7H5R10Z25dpw6** | 6 |
| **E29H14R13Z47dpw3** | 8 |
| **E15M7R10Z25dpw7** | 9 |
| **M9H14R10Z34dpw3** | 10 |
| **H6R10Z28dpw7** | 11 |
| **E16H6R9Z24dpw3** | 12 |
| **H16R12Z49dpw3** | 13 |
| **P2M7Z27dpw7** | 13 |
| **E16H6R10dpw7** | 15 |
| **E25H6R10dpw7** | 15 |
| **E25H10R10dpw7** | 15 |
| **H6dpw7** | 18 |
| **E10H6R6dpw7** | 19 |
| **E25R23.5dpw7** | 20 |
| **E40H15R10dpw2** | 21 |
| **E25R11dpw7** | 22 |
| **E45R23.5dpw2** | 22 |
| **E6H6dpw7** | 24 |
| **E15H6dpw7** | 24 |
| **E25H6dpw7** | 24 |
| **E25H10dpw7** | 24 |
| **E90R23.5dpw1** | 24 |
| **E90R24Z65.5dpw1** | 24 |

**Supplementary Table 10: Clinical rankings from [2]**

| **GranSim Name** | **Rank (based on culture (%))** |
| --- | --- |
| **E15M7R10Z25dpw7** | 1 |
| **P2M7Z27dpw7** | 2 |
| **E16H6R10Z27dpw7** | 3 |
| **E25H6R10Z33dpw7** | 3 |
| **M7H5R10Z25dpw6** | 5 |
| **M9H14R10Z34dpw3** | 5 |
| **E18H5R10p25Z5dpw5** | 7 |
| **H6R10Z28dpw7** | 8 |
| **H16R12Z49dpw3** | 9 |
| **E29H14R13Z47dpw3** | 10 |
| **E17.5H5R10Z25dpw6** | 11 |
| **E25R23.5dpw7** | 12 |
| **E45R23.5dpw2** | 13 |
| **E90R23.5dpw1** | 13 |
| **E90R24Z65.5dpw1** | 13 |
| **E16H6R9Z24dpw3** | 16 |
| **E25H6R10dpw7** | 17 |
| **E40H15R10dpw2** | 18 |
| **H11R10dpw5** | 19 |
| **E25R11dpw7** | 20 |
| **E10H6R6dpw7** | 21 |
| **E16H6R10dpw7** | 22 |
| **R12.5dpw7** | 23 |
| **E6H6dpw7** | 23 |
| **H6R11dpw7** | 23 |
| **E25H6dpw7** | 26 |
| **E15H6dpw7** | 27 |
| **E25H10dpw7** | 28 |
| **H6dpw7** | 29 |
| **E25H10R10dpw7** | 30 |

**Supplementary Table 11: Cmax values for each drug**

| **Drugs** | **Cmax (mg/L)** |
| --- | --- |
| INH | 0.8 |
| RIF | 4 |
| PZA | 50 |
| EMB | 8 |
| MXF | 15 |
| BDQ | 250 |
| PTM | 12 |
| LZD | 60 |

**References**

1. Budak M, Via LE, Weiner DM, Barry CE, 3rd, Nanda P, Michael G, et al. A systematic efficacy analysis of tuberculosis treatment with BPaL-containing regimens using a multiscale modeling approach. CPT Pharmacometrics Syst Pharmacol. 2024;13(4):673-85. Epub 20240226. doi: 10.1002/psp4.13117. PubMed PMID: 38404200; PubMed Central PMCID: PMCPMC11015080.

2. Bonnett LJ, Ken-Dror G, Koh GCKW, Davies GR. Comparing the Efficacy of Drug Regimens for Pulmonary Tuberculosis: Meta-analysis of Endpoints in Early-Phase Clinical Trials. Clinical Infectious Diseases. 2017;65(1):46-54. doi: 10.1093/cid/cix247.
